# Variational Phylodynamic Inference Using Pandemic-scale Data

**DOI:** 10.1093/molbev/msac154

**Published:** 2022-07-11

**Authors:** Caleb Ki, Jonathan Terhorst

**Affiliations:** Department of Statistics, University of Michigan, Ann Arbor, MI, USA; Department of Statistics, University of Michigan, Ann Arbor, MI, USA

**Keywords:** phylogenetics, phylodynamics, birth-death model, pandemic-scale

## Abstract

The ongoing global pandemic has sharply increased the amount of data available to researchers in epidemiology and public health. Unfortunately, few existing analysis tools are capable of exploiting all of the information contained in a pandemic-scale data set, resulting in missed opportunities for improved surveillance and contact tracing. In this paper, we develop the variational Bayesian skyline (VBSKY), a method for fitting Bayesian phylodynamic models to very large pathogen genetic data sets. By combining recent advances in phylodynamic modeling, scalable Bayesian inference and differentiable programming, along with a few tailored heuristics, VBSKY is capable of analyzing thousands of genomes in a few minutes, providing accurate estimates of epidemiologically relevant quantities such as the effective reproduction number and overall sampling effort through time. We illustrate the utility of our method by performing a rapid analysis of a large number of SARS-CoV-2 genomes, and demonstrate that the resulting estimates closely track those derived from alternative sources of public health data.

## Introduction

The COVID-19 pandemic has demonstrated an important supporting role for phylogenetics in epidemiology and public health, while also creating unforeseen technical and methodological challenges. As the first global public health event to occur in the era of ubiquitous sequencing, the pandemic has resulted in a data explosion of unprecedented proportions. GISAID, a worldwide repository of SARS-CoV-2 genomic data, currently has over 7.5M samples, with contributions from almost every country (Elbe and Buckland-Merrett 2017; van Dorp et al. 2021). A phylogenetic representation of this database is believed to be the largest ever constructed (Turakhia, Thornlow, Hinrichs, De Maio, et al. 2021). Existing phylogenetic methods, which were developed and tested on datasets orders of magnitude smaller, are inadequate for pandemic-scale analysis, resulting in missed opportunities to improve our surveillance and response capabilities (Hodcroft et al. 2021; Morel et al. 2021; Ye et al. 2021).

These shortcomings have spurred new research initiatives into phylogenetic inference methods capable of analyzing millions of samples. In particular, there has been significant recent progress in estimating and/or placing novel sequences onto very large phylogenies (Minh et al. 2020; Aksamentov et al. 2021; Turakhia, Thornlow, Hinrichs, De Maio, et al. 2021; Ye, Shum, et al. 2022; Ye, Thornlow, et al. 2022). Accurate estimation of the underlying phylogeny has numerous downstream applications, including contact tracing (e.g., Lam-Hine et al. 2021; McBroome et al. 2022), surveillance (e.g., Abe and Arita 2021; Klink et al. 2021), and improved understanding of pathogen biology (e.g., Majumdar and Sarkar 2021; Turakhia, Thornlow, Hinrichs, Mcbroome, et al. 2021).

Another area of active research in phylogenetics, distinct from tree inference, is so-called *phylodynamics*, which seeks to understand how immunological, epidemiological, and evolutionary forces interact to shape viral phylogenies (Volz et al. 2013). Here, the quantity of interest is typically a low-dimensional parameter vector characterizing the underlying phylodynamic model, whereas the phylogeny itself is a nuisance parameter. Of particular interest for the current pandemic are methods that can estimate effective population size and reproduction number of the pathogen from viral genetic data (e.g., Lai et al. 2020; Zhou et al. 2020; Campbell et al. 2021; Volz et al. 2021). Compared to phylogeny estimation, less progress has been made on so-called “phylodynamic inference” at the pandemic scale. This absence motivates the present study.

Bayesian methods are often preferred for phylodynamic inference because, in complex datasets, there are many possible trees which explain the data equally well. Hence, downstream quantities of interest possess a potentially significant amount of “phylogenetic uncertainty” which is not reflected in frequentist point estimates. Unfortunately, Bayesian phylogenetic procedures inherently scale very poorly: the space of phylogenetic trees grows rapidly, and there are an astronomical number of possible trees to consider, even for relatively small samples. Consequently, on large problems, the workhorse algorithm of field, Markov chain Monte Carlo (MCMC), tends to either conservatively explore very limited regions of tree space, or liberally propose large moves that are often rejected (Whidden and Matsen 2015; Zhang and Matsen 2019).

Even before the pandemic, awareness of the scalability issues surrounding Bayesian phylogenetics was growing (Höhna and Drummond 2012; Whidden and Matsen 2015; Aberer et al. 2016; Dinh et al. 2017). As a scalable alternative to MCMC, variational inference (VI) has recently garnered some attention in phylogenetics. VI is a general method for sampling approximately from a posterior distribution using techniques from optimization (Jordan et al. 1999). Fourment et al. (2020) used VI to accelerate computation of the marginal likelihood of a fixed tree topology. Fourment and Darling (2019) used the probabilistic programming language STAN to perform variational inference of the Bayesian skyline model (Pybus et al. 2000). Both of the preceding methods only analyze a fixed tree topology, so they cannot account for phylogenetic uncertainty. Simultaneously, Zhang and Matsen (2018, 2019) and Zhang (2020) have made progress on a full variational approach which includes optimization over the underlying topology. Although these innovations represent significant advances in terms of performance, they still cannot come close to exploiting all of the information contained in a pandemic-scale data set.

## New Approaches

Inspired by these works, and responding to the need for better tooling to study the ongoing pandemic, we devised a method capable of providing accurate and calibrated estimates of the rates of transmission and recovery for COVID-19 using data from tens of thousands of viral genomes. Our approach unites several threads of research in phylogenetics and scalable Bayesian inference. We build on aforementioned advances in variational phylogenetic inference (Fourment and Darling 2019; Zhang 2020), as well as recent progress in phylodynamic modeling of infectious diseases (Stadler et al. 2013), Bayesian stochastic optimization (Hoffman et al. 2013), and differentiable programming (Bradbury et al. 2018). To achieve this level of scalability, our method makes several tradeoffs and approximations which are detailed below. Briefly, we adopt a divide-and-conquer strategy where distant subtrees of a very large phylogeny are assumed to evolve approximately independently, and we further assume that topological estimates of these subtrees are an accurate reflection of their distribution under the prior. We argue that these are reasonable approximations in the context of an massive, global phylogeny, and that their combined effect appears to be benign: the resulting estimates closely agree with the existing state of the art on simulated data, and exhibit a remarkable level of concordance with ground-truth estimates on real data, although taking just minutes to produce.

## Results

In this section, we test our method on both simulated and real data, and compare it to the existing implementation of the birth–death skyline model in BEAST.

### Simulation

First, we performed a simulation study to evaluate how well VBSKY approximates the posterior distribution compared with BEAST. We studied four different scenarios:

1. Constant: the effective reproductive number stays constant through time.
2. Decrease: there is a sharp drop in the effective reproductive number.
3. Increase: there is a sharp increase in the effective reproductive number.
4. Zigzag: the effective reproductive number goes through a series of decreases and increases.

We simulated transmission trees using the R package TreeSim (Stadler 2011) and generated sequences data along each tree using the program Seq-Gen (Rambaut and Grass 1997).

Across all scenarios, the rate of becoming uninfectious, δ is held constant at δ(t)=4 for all t. The sampling rate is also held constant at s(t)=0.25. Only R is allowed to vary. Under the constant scenario, R(t)=1.3 for all t. In the decrease scenario,

$$R(t)=\left\{ \begin{array}{c} 2.25,\;t\leq 1\\ 0.75,\;t>1. \end{array}\right.$$

In the increase scenario,

$$R(t)=\left\{ \begin{array}{c} 1,\;t\leq 3\\ 2.5,\;t>3. \end{array}\right.$$

In the zigzag scenario,

$$R(t)=\left\{ \begin{array}{c} 2.0,\;t\in [0,1]\cup (2,3]\\ 0.75,\;t\in (1,2]\cup (3,4]. \end{array}\right.$$

Each simulation was run for four time units, and ten trees were generated under each scenario. Because the sampling process is stochastic in this model, the size of the simulated tree varied from run to run. The minimum (maximum) number of samples in each under the constant, decrease, increase, and zigzag scenarios was 175 (1553), 117 (590), 124 (1075), and 161 (1852), respectively.

We compared the performance of our method with the current state-of-the-art method for Bayesian phylogenetic analysis (BEAST; Bouckaert et al. 2019). BEAST allows for the birth–death skyline model to be used as a tree prior, facilitating direct comparison with VBSKY. Because BEAST uses MCMC to estimate the posterior, the number of sequences it can analyze is limited. Therefore, for each simulation, we randomly sampled 100 sequences for BEAST to analyze. We allowed BEAST to run long enough that the effective sample size exceeded 1,000 for each evolutionary parameter. Since VBSKY is not limited by sample size, we analyzed all sequences in each simulation, as follows: We set the size of each random subsample to be b=100 tips. The number of trees in the ensemble was set to be the smallest integer such that the number of trees multiplied by 100 was larger than the number of sampled sequences. Under this scheme, each sequence was sampled approximately once on average.

**Fig. 1.**
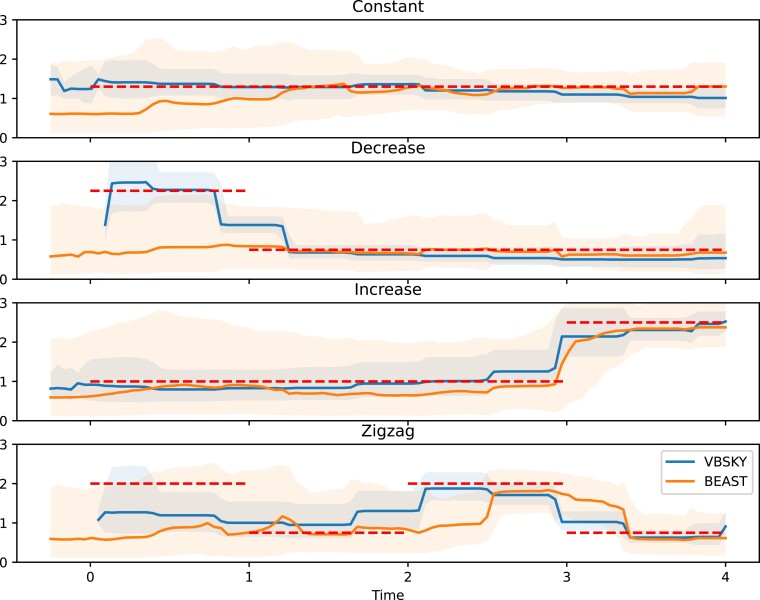
Median of the medians and the equal-tailed 95% credible intervals of the posteriors of the effective reproductive number over time of the 10 simulations for each scenario using VBSKY and BEAST. The dotted line is the true effective reproductive number over time.

The results of the simulation study are shown in figure 1, which displays the median of the medians and 95% equal-tailed credible intervals of the simulations under each scenario using VBSKY and BEAST. In the constant and increase scenarios, both BEAST and VBSKY adequately capture the true value of the effective reproductive number. However, in the decrease and zigzag scenarios, only VBSKY is able to capture the initial elevated effective reproductive number further back in time at the start of the simulation. In contrast, BEAST appears to revert to the prior as it seems unable to detect transmission events within those intervals. Because VBSKY allows for more sequences to be analyzed, it is able to detect transmission events further back in time. The credible intervals given by BEAST are wider than those of VBSKY, and do a better job of covering the true model in some cases; we return to this point in section “Discussion.”

Even though in some cases we analyzed hundreds more sequences using VBSKY than when we used BEAST, the run-time of VBSKY was 71.75 s on average for each simulation, whereas BEAST took 20 min to perform $10^{7}$ MCMC steps. The simulation results show that VBSKY produces comparable results to BEAST in less time, and in some cases it is more accurate as well.

As an additional point of comparison, we also analyzed the smaller data sets given to BEAST using VBSKY. In this case, we again set the size of each random subsample to be 100, and only use a single tree. The results are displayed in supplementary figure S8, Supplementary Material online. Using less data, VBSKY provides similar albeit slightly less accurate results. It is still able to correctly infer changes in the effective reproductive number even in the cases where BEAST is unable to using the same dataset. The difference in accuracy between using the smaller or full datasets is most pronounced in the increase and zigzag scenarios, where VBSKY is not able to accurately capture the magnitude of the increase in the effective reproductive number. The results from this analysis suggest that although VBSKY needs a large sample for optimal performance, it can perform about as well as BEAST using a comparable amount of data.

### Analysis of the Global Pandemic

We tested our method on a large, serially sampled COVID-19 dataset from the GISAID initiative (Elbe and Buckland-Merrett 2017). At the time this analysis was performed, there were 6.5M SARS-CoV-2 sequences in the database. In addition to the raw nucleotide data, GISAID provides sample time and location information. The collection dates of the sequences range from January 3, 2020 to December 8, 2021.

For our analysis, we chose four geographical study areas: the states of Michigan and Florida, as well as aggregate data for the entire USA and UK. It is important to study the epidemiology of COVID-19 at the sub-national level as many public health policies such as mask mandates, stay at home orders, vaccine distribution, and other social distancing measures are enforced at the state level. Policies or decisions made in one state may not be detected studying national data. Due to the differences in health policies across states and the reduced frequency of travel during the pandemic, we expect the incidence and prevalence of COVID-19 to vary from state to state. On the other hand, policies are sometimes made at the national level, and more recently travel especially around the holidays has become widespread, so understanding trends at a national level is equally vital. It is also interesting to compare the epidemiology of the pandemic in the USA and UK, as the two countries are demographically similar, but differ widely in terms of their healthcare systems, governance, and policy responses (Unruh et al. 2022).

After filtering the sequences by location, the number of sequences were 81,375, 34,978, 1,280,563, and 1,143,909 for Florida, Michigan, the USA, and the UK, respectively. We noticed that the number of confirmed cases increased or decreased based on the day of the week, likely because fewer cases are reported over the weekend. To correct for any inaccuracies in the sample time distribution, we set all sequences sampled in the same calendar week to have the same sample time. We used a fixed molecular clock model with substitution rate $1.12\times 10^{-3}$/bp/year, as estimated by the World Health Organization (WHO) (Koyama et al. 2020). We compared our estimates with a “ground truth” estimator of the effective reproductive number which is derived from orthogonal (i.e., non-genetic) public health data sources (Shi et al. 2021).

**Fig. 2.**
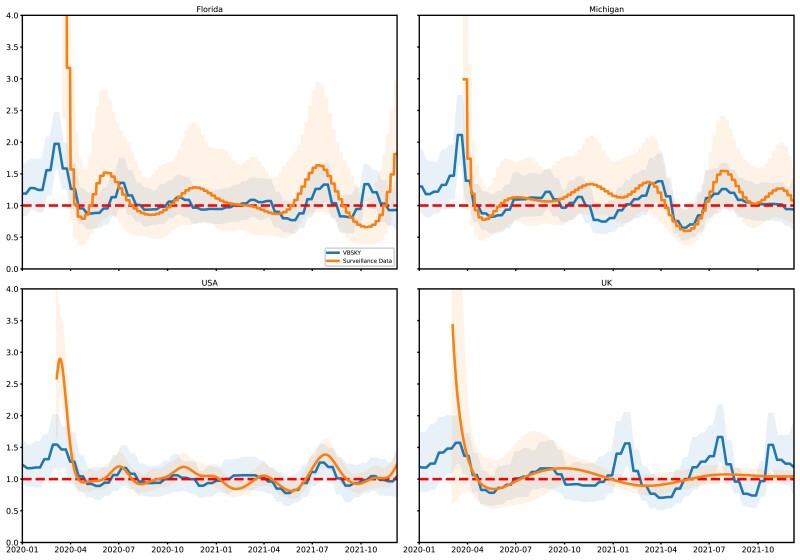
Posterior of R for Florida, Michigan, and the USA using biased sampling and a strong prior on s. For each method the posterior median and equal-tailed 95% credible interval are shown. The dotted line is R=1.

We experimented with several different configurations for the various hyperparameters supported by our method. The prior and hyperprior settings for all of the scenarios described below are shown in table 1. In general, the three tuning parameters of VBSKY that had the biggest effect on its output were the level of smoothing, as specified by the precision hyperparameter on the Gauss–Markov random field (GMRF) smoothing prior (columns $\tau_{R}$ and $\tau_{s}$ in table 1; see also section “Model Parameterization”); the position of the origin (column $x_{1}$ in the table); and the strategy used to generate the ensemble of sampled subtrees (cf. section “Scalable Inference” and supplementary section S3, Supplementary Material online). Figure 2 and supplementary figure S1, Supplementary Material online showcase the best estimates that we obtained for R and s, respectively, after hyperparameter tuning; results for some other choices are shown in supplementary figures S2–S7, Supplementary Material online. We first discuss the qualitative features of these estimates, and then explain how we selected the hyperparameters.

**Table 1.** Prior Distributions Used in Analyses.

Analysis	R	s	$\tau_{R}$	$\tau_{s}$	$x_{1}$
Uninformative Smoothing	LogN(1,1)	Beta(0.02, 0.98)	Gamma(0.001, 0.001)	Gamma(0.001, 0.001)	LogN(−1.2, 0.1)
Less Smoothing	LogN(1,1)	Beta(20, 980)	Gamma(10, 100)	Gamma(10, 100)	LogN(−1.2, 0.1)
Biased/Cluster Sampling	LogN(1,1)	Beta(20, 980)	Gamma(0.001, 0.001)	Gamma(0.001, 0.001)	—
Multistrain	LogN(0,1)	Beta(2, 98)	Gamma(10000, 0.01)	Gamma(10000, 0.01)	—

In general, figure 2 shows a surprisingly close match between our model output and the ground-truth, which we reiterate was estimated using a completely different type of data. As already noted when we compared VBSKY with BEAST, the credible bands produced by VBSKY tend to be narrower. This could reflect differences in the underlying data, or violations of the modeling assumptions described in section “Materials and Methods.” Interestingly, both methods appear unable to reject the null hypothesis R=1 except for very early in the pandemic (winter 2020) and very recently (spring–summer 2021). The largest difference between the VBSKY and public health-derived estimates are observed for the UK; the latter are much smoother and do not exhibit pronounced spikes compared with the former. However, the VBSKY estimates are strikingly concordant with the macro-scale history of the COVID-19 pandemic in the UK, which consisted of a first wave in January–May 2020; a second wave which began in September 2020, abated in the late fall, and peaked in January 2021; and Delta- and Omicron-fueled waves which peaked in July and November 2021, respectively (du Plessis et al. 2021; Sutherland et al. 2021; UK Health Security Agency 2022). VBSKY recapitulates these dynamics almost exactly. We hypothesize that estimates for the UK may be more accurate because of greater uniformity in the collection and reporting of COVID-19 genetic data by the UK National Health Service compared with the health care system in the USA.

In order to obtain these estimates, we utilized a “biased” sampling approach whereby we preferentially sampled leaves in the infection tree which occurred in the distant past, in order to give our method better power to infer epidemiological history there. Increases in our testing capacity over time cause the overall density of sample times to skew heavily towards the recent past (supplementary fig. S9, Supplementary Material online). Hence, sampling infections uniformly at random causes our method to have good power to infer the recent epidemiological history of the pandemic, at the expense of poor resolution in the early phases. Indeed, this is exactly what we observed when we re-ran our method using this type of sampling strategy (supplementary fig. S2, Supplementary Material online). Except for Michigan, where sampling has been relatively more uniform over time, the posterior for R is very flat further back in the past; the posterior distribution is essentially that of the prior in this region.

We also studied whether it was possible to obtain good estimates of R using a combination of uniform sampling and decreased smoothing. Supplementary figure S3, Supplementary Material online shows the posterior when we set the prior of the smoothing parameter to be a gamma distribution with a=10 and b=100, giving a mean of 0.1 and variance 0.001. Looking at the top left panel (Florida) of supplementary figure S3, Supplementary Material online, we see that the posterior median of R for VBSKY is no longer flat and instead oscillates slightly to better match the results using surveillance data. The bottom left panel (USA) also shows the estimates for R for the entire USA are also no longer completely flat further back in the past. The top right panel (Michigan) shows that even with less smoothing, the results for VBSKY in Michigan match well with the surveillance data. When the sample time distribution is unbalanced, as with Florida and the USA, imposing less smoothing can help better capture the signal where the sampling may be more sparse. However, it also widens the credible intervals. This is not universally true however as looking at the bottom right panel (UK), whereas the estimates for R are not completely flat, given what supplementary figure S10, Supplementary Material online tells us about case count, we would expect larger peaks for R over time.

Finally, we experimented with a cluster-based sampling approach, whereby we selected random subclades from a pre-estimated SARS-CoV-2 phylogeny (Lanfear 2020). Specifically, we sampled random tips within each study region, and then successively “walked” up the tree until reaching an ancestral node which subtended at least 200 leaves. Each subsample is then made up of a single cluster. Other hyperparameter settings were the same as in the “Biased sampling” scenario. Results of this experiment are shown in supplementary figures S4 and S7, Supplementary Material online. The results are generally similar to the uniform sampling strategy—there is fairly good power to estimate R in the recent past, but estimates in the distant past appear somewhat oversmoothed.

Overall, using less smoothing only (supplementary fig. S3, Supplementary Material online), VBSKY was able to capture the shape of estimates using surveillance data, but the biased/stratified sampling approach results in a much closer estimate of R further back in the past. One drawback of stratified sampling is that the estimates of R towards the present seem to be further away from the estimates using surveillance data. Hence, although nonuniform sampling can improve estimates within time periods where sampling is sparse, it can also bias them in densely sampled regions.

The other hyperparameters were chosen as follows: we deterministically fixed the origin to 0.3 years prior to the earliest sample date (therefore, no prior on $x_{1}$ is listed in the table). We encountered occasional numerical issues when attempting to learn the variational posterior distribution over the origin parameter. This was not entirely unexpected since there is only weak power to infer the origin time using this model (Stadler et al. 2013). We ran VBSKY with 50 subsamples of 200 sequences for a total of $10^{4}$ sequences. Additional discussion of the effect various hyperparameters on our method’s output can be found in supplementary section S-4, Supplementary Material online.

#### Comparison to BEAST

We ran BEAST on the same data set as in the previous section. BEAST was incapable of analyzing the same number of samples as VBSKY, so to facilitate comparison, we limited the number of sequences we analyzed with BEAST. Both the sample size and the sampling scheme can affect the results of the analysis as well as the mixing time, so we compared how BEAST performed with different combinations of sample sizes and sampling schemes. We ran BEAST with both 100 and 500 sequences. For each sample size, we sampled the most-recent sequences by date (contemporary sampling), and we also sampled uniformly at random without any regard to the sample time (random sampling). The XML configuration files we used to run BEAST are included in the supplementary data.

Even after greatly reducing the number of sequences analyzed, accurately sampling from the posterior may still take longer than using VBSKY. We performed both a “short” run for BEAST, where the MCMC sampler is only allowed to run for as long as it took VBSKY to analyze the full data, as well as a “long” run where BEAST was allowed to perform 100 MCMC million iterations, or run for 24 h, whichever was shorter.

The estimates of the effective reproductive number of the short and long runs are shown in supplementary figures S11–S14 and S15–S18, Supplementary Material online, respectively. For the short runs, depending on the number of samples and the sampling scheme, the results varied widely. Under a short time constraint, the posteriors using 500 tips and the random sampling scheme for Florida, the USA, and the UK as well as 500 tips and both sampling schemes for Michigan were mostly flat and centered close to 1. The posteriors did not reflect the rise and fall in R that is exhibited in both the surveillance data and VBSKY estimates. In most cases, BEAST is unable to capture any signal further back in the past, and the posterior provided by BEAST does not track the estimates provided by the surveillance data as well as VBSKY.

In the long runs, the issue of completely flat posteriors when using 500 tips mostly disappeared. However, BEAST is only capable of producing comparable results to VBSKY and the surveillance method when analyzing 100 tips sampled uniformly at random, presumably because mixing occurred more rapidly in the time allotted. The long runs also illustrate that uniform random sampling performs better than most-recent sampling when running BEAST. This indicates that having samples throughout time may help infer more transmission events further back in the past rather than having only contemporary sequences. The discrepancy between using 100 tips and 500 tips exists only when the sampling scheme is random. When using contemporary sequences, BEAST is able to complete 100 million iterations. But when random sampling is used, because the MCMC sampler mixes more slowly, BEAST was unable to complete 100 million MCMC moves within 24 h.

In summary, BEAST performed fairly well when we randomly sampled 100 tips, though there was considerable variation between data sets and scenarios. The main difference between VBSKY and BEAST is that the latter was usually unable to capture signal far back in the past. Analyzing more sequences could help, but the computational difficulties that would ensue imply that it is not practical to completely resolve this issue if time is a constraint. Overall, our results indicate that efficiently analyzing thousands of sequences, even using an approximate inference method, generally leads to a sharper posterior which is closer to the ground truth.

#### Strain Analysis

A distinct advantage of the molecular approach to epidemiological inference is the ability to incorporate genetic signals which do not exist in traditional surveillance data. As an example of this strategy, we used our method to study the history of individual COVID-19 variants. Using the variant annotations provided by GISAID, we split the data into subsets containing Alpha, Delta, and Omicron samples for each of the four study regions described above. To generate ensembles of subtrees for our method, we randomly sampled subtrees from a pre-computed reference phylogeny (Lanfear 2020). We also found it necessary to make some adjustments to the priors used the previous section. Specifically, given that we are examining three variants which successively replaced each other, a prior of R>1 is not necessarily appropriate, and we found that results were improved if we decreased the prior mean of R. (We discuss this choice further below.) Also, for the GMRF smoothing prior, we chose $\tau_{R}$ and $\tau_{s}$ to have large expectations to increase smoothing.

**Fig. 3.**
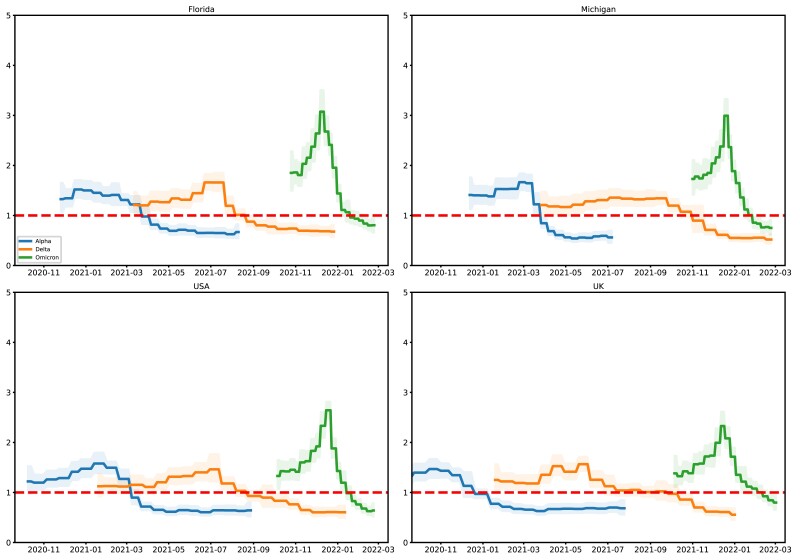
The posterior median and equal-tailed 95% credible interval of R for the Alpha, Delta, Omicron variants.

The results of our analysis are shown in figure 3 for R (supplementary fig. S19, Supplementary Material online for s). The Alpha variant of COVID-19, also known as lineage B.1.1.7, originated in England and was first reported in the USA in early 2021. Using surveillance data, Volz et al. (2021) showed that at the time, the Alpha variant had a transmission advantage over other variants, which is why it came to dominate in the USA and UK in early 2021. There are no samples for the Alpha variant beyond summer 2021, so the estimates for Alpha are truncated at various points during that period depending on the region considered. As shown in supplementary figure S10, Supplementary Material online, the number of cases was dropping in the regions after the first third of the year, corresponding to a decrease in R below one for the Alpha variant. At the same time, the Delta variant rose in prevalence, such that R is estimated greater than one in all cases until about the third quarter of 2021. Finally, Fall 2021 saw the emergence of the Omicron variant, which quickly rose in prevalence until it was the dominant strain. Estimates of R across all study regions peak around November or December 2021, before declining rapidly; by March 2022, the R value of Omicron is declining estimated less than 1 in except perhaps in the UK. Of the three variants, Omicron is estimated to have the highest peak R value in all regions, likely reflecting its increased transmissibility.

Finally, we also explored whether the use of a different method for generating ensembles of tree topologies (sUPGMA; see supplementary section S2, Supplementary Material online) had any effect on our results (supplementary figs. S20 and S21, Supplementary Material online). We found that results were generally consistent across the two methods, however the estimates obtained using sUPGMA indicated slightly different dynamics for the Omicron variant in the UK in the early portion of 2022—instead of R<1, the sUPGMA-derived estimates showed that Omicron continued to expand in the UK throughout Winter 2022.

Analysis of the sampling fraction over time (supplementary fig. S19, Supplementary Material online) also shows some interesting trends, for example sampling of the Alpha variant in Michigan seems to have been high compared with other areas and strains, whereas sampling of the Delta variant was rather low. Another interesting result is the apparent divergence in R for the Omicron variant between the USA and UK in the beginning of 2022. For Michigan and Florida, as well as the USA as a whole, R is estimated to have dropped below 1 around January 2022, and the credible intervals contain R=1. In contrast, R in the UK continued to climb throughout the winter, and is credibly different from 1 as recently as March 2022.

Finally, we also explored using other hyperparameter settings to analyze these data, but found that they produced generally worse results. In particular, without additional smoothing, our model unrealistically estimated large oscillations in R, especially for the Omicron variant. Additionally, we noticed that for the Alpha variant, since the number of available samples drops precipitously near the point of truncation, the prior distribution dominated the posterior in the recent past, which caused R to counterintuitively increase in the direction of the prior mode (as well as widening the credible band). Since R>1 is not a reasonable prior assumption for a strain which is known to have vanished, we shrank the prior distribution towards zero to attenuate this effect. We also found that increased smoothing also helped mitigate this issue, as intervals with a low number of samples are more heavily influenced by neighboring intervals.

## Discussion

In this paper, we presented the variational Bayesian skyline, a method designed to infer evolutionary models from large phylogenetic datasets. Our method works by fitting a variational Bayesian posterior distribution to a certain approximation of the phylogenetic birth–death model. We showed that, under some simplifying heuristic assumptions, it can be used to infer epidemiologically relevant quantities such as the effective reproduction number and sampling fraction. We demonstrated that our estimates adhere reasonably closely to those formed using MCMC, but are much faster to obtain, and able to incorporate larger numbers of observations. On real data, we showed how our model corroborates public health surveillance estimates, and could work to fill in knowledge gaps when such data are unavailable.

The improvement in speed of our model compared to previous approaches is due to both the divide and conquer strategy and the stochastic variational inference component. The divide and conquer strategy obviates the need to estimate large phylogenies, whereas still retaining information from a large number of samples. In turn, this reduces the number of nuisance parameters (e.g., branch lengths) that we must coestimate along with the epidemiological parameters, and also reduces the computational burden of using expensive tree inference algorithms. However, the divide and conquer strategy would not be possible without the use of stochastic variational inference, as MCMC is prohibitively slow even for small samples. Hence, an MCMC-based divide and conquer strategy method would still be unable to incorporate large numbers of sequences. *Both* stochastic variational inference and the divide and conquer strategy are necessary for our approach to work.

One shortcoming of our model is that it tends to be overconfident, in the sense that it produces credible intervals which are narrower compared to other methods, and not as well calibrated in simulations. Generally, it is preferable for a method to overcover since this is inferentially more conservative. We believe this behavior is attributable to the heuristics that underlie our approach: since they ignore certain forms of dependence in the data, they create the illusion of a larger sample size than actually exists. We suggest that the credible intervals produce by our method are best interpreted relatively, as showcasing portions of time where the estimates are particularly tight or loose.

Our method could be extended in several ways. Currently, it estimates the tree topology and the continuous variables separately, relying on a distance-based method to infer the topology. While faster, distance-based methods are less accurate than likelihood-based methods for tree reconstruction (Kuhner and Felsenstein 1994). Our method could be potentially extended to unify the estimating procedure for tree topologies and other variables under one variational framework allowing (Zhang and Matsen 2019). We also take random subsamples of data to accelerate our inference. However, the subsampling approach we adopt is naive, and future work could include developing an improved strategy for subsampling in phylogenetic problems.

The variational inference scheme we used makes a standard but highly simplified mean-field assumption about the dependence structure of the variational approximating family. We also experimented with other, recent approaches such as normalizing flows (Rezende and Mohamed 2015), but observed that, consistent with earlier findings (Fourment and Darling 2019), they did not measurably improve the results and occasionally caused the algorithm to fail to converge. If our approach is adapted to more complex problems, it could be advantageous to revisit this modeling choice.

Currently, our method is restricted to using a strict molecular clock model. Additionally, the substitution models in our method do not currently allow for rate heterogeneity across sites. Allowing for more flexible and complex substitution and clock models could aid in the application of our method to other data sets that evolve differently than COVID-19, when the time scale of the epidemic is much larger. Lastly, we use a GMRF prior on the rate vector parameters. Other choices of prior based on Gaussian processes (Palacios and Minin 2012, 2013) or some other nonparametric smoother (e.g., Faulkner and Minin 2018) could lead to improved estimates in more complex scenarios.

## Materials and Methods

In this section, we derive our method, which we call variational Bayesian skyline (VBSKY). As the name suggests, VBSKY descends from a lineage of earlier methods designed to infer evolutionary rate parameters from phylogenetic data (Pybus et al. 2000; Drummond et al. 2005; Minin et al. 2008; Gill et al. 2013). Our running example will be inferring the epidemiological history of the COVID-19 pandemic, but the method applies generally to any evolving system that is aptly modeled using a phylogenetic birth–death or coalescent process and approximately meets the assumptions described below.

### Notation and Model

The data consist of a matrix of aligned sequences $D=\{A,C,G,T,N{\}}^{n\times L}$, where n is the number of viral sequences and L is the number of sites, and a vector of times when each sample was collected $y=(y_{1},\ldots ,y_{n})$ where $y_{1}\leq \cdots \leq y_{n}$. Row j of D corresponds to a sequenced viral genome collected from an infected host at time $y_{j}$. Subsamples of *rows* of D are denoted by $D_{i}\in \{A,C,G,T,N{\}}^{b\times L}$, with corresponding sample times $y^{\left(i\right)}=(y_{1}^{\left(i\right)},\ldots ,y_{b}^{\left(i\right)})$, where b is the size of the subsample. We occasionally abuse notation and write $D_{i}\subset D$ to denote a subsample, and |D| to denote the number of samples contained in a dataset (so e.g., $|D_{i}|=b$ above). Phylogenetic trees are denoted by $T=(T^{\mathrm{topo}},T^{\mathrm{br}})$, which we decompose into a discrete topological component and continuous branch length component. Given n sampled taxa, the topological component $T^{\mathrm{topo}}$ lives in the space of rooted, labeled bifurcating trees on n leaves, and the branch length component lives in the non-negative orthant $R_{\geq 0}^{2n-1}$ and gives the length of each edge of the tree (including an edge from crown to origin).

The data are assumed to be generated according to a phylogenetic birth–death skyline model (Nee et al. 1994; Morlon et al. 2011). In this model, samples are related by an unobserved “transmission tree” that records every infection event that occurred during the pandemic. Leaf nodes in the transmission tree represent sampling events, and internal nodes represent events where the virus was transmitted from one host to another. Edges denote periods during which the virus evolved within a particular host, with the length proportional to the amount of evolutionary time that elapsed between the parent and child nodes. The distribution of the infection tree depends on three fundamental parameters, usually denoted by μ(t), λ(t), and ρ, which are respectively the time-varying per-capita rates at which extant lineages in the phylogeny go extinct and speciate, and the fraction of the extant population that was sampled at the present.

Further generalizations (Stadler et al. 2013) incorporate both random and deterministic sampling across time, and it was also shown how phylogenetic BD model can be used for parameter estimation in the susceptible-infected-recovered model (Kermack and McKendrick 1927) that forms the foundation of quantitative epidemiology. Let ψ(t) denote the rate at which each extant lineage is sampled in the phylogeny. (Henceforth we suppress dependence on time, but all parameters are allowed to be time-varying.) If we assume that sampling is tantamount to recovery (a valid assumption when positive testing leads to quarantine, as is generally the case during the current pandemic), then the overall rate of becoming uninfectious is δ=μ+ψ; the average time to recovery is 1/δ; the sampling proportion is s=ψ/δ; and the effective reproduction number is R=λ/δ. Using prior knowledge, it is also common to specify an origin time $t_{0}$ when the pandemic began.

Let $\zeta =(R,\delta ,s,t_{0})$ denote the vector of epidemiological parameters of interest. The hyperprior on ζ is denoted π(ζ). The latent transmission tree describing the shared evolutionary history of all of the sampled pathogens is denoted by $T=(T^{\mathrm{topo}},T^{\mathrm{br}})$. We assume a simple “strict clock” model, with known rates of substitution, so that no additional parameters are needed to complete the evolutionary model.

We desire to sample from the posterior distribution of ζ given the phylogenetic data set D. Let p(T∣ζ) denote the likelihood of the transmission tree given the evolutionary model. An expression for p(T∣ζ) can be found in Stadler et al. (2013, Theorem 1), and is reproduced in supplementary Appendix S-1, Supplementary Material online for completeness. The data depend on ζ only through T, so that p(D∣T,ζ)=p(D∣T). Here p(D∣T) denotes the “phylogenetic likelihood,” which can be efficiently evaluated using the pruning algorithm (Felsenstein 1981). Putting everything together, the posterior distribution over the unobserved model parameters is

(1) p(ζ,T∣D)∝p(D∣T)p(T∣ζ)π(ζ).

### Scalable Inference

The constant of proportionality in (1) is p(D), the marginal likelihood after integrating out all (hyper)parameters and the unobserved tree T. In large phylogenetic data sets, exact evaluation of the marginal likelihood is impossible due to the need to enumerate all possible trees, a set whose cardinality explodes in the number of taxa (Alfaro and Holder 2006). In practice, methods such as Markov chain Monte Carlo (e.g., Drummond and Rambaut 2007) which do not require evaluating p(D) are utilized.

Since current phylogenetic MCMC algorithms cannot scale up to pandemic-sized datasets, we propose to modify the inference problem (1) using a few heuristics in order to make progress. Let $D_{1},D_{2},\ldots ,D_{S}\subset D$ be subsamples of $b_{1},\ldots ,b_{S}$ rows from the full dataset. If the subsamples are temporally and geographically separated, and $b_{i}\ll n$, then it is reasonable to suppose that these subsamples are approximately independent conditional on the underlying evolutionary model.

Heuristic 1. *In a very large phylogenetic dataset D, small subsets $D_{1},D_{2}\subset D$ with $\left|D_{1}|,|D_{2}|\ll |D\right|$ that are sufficiently separated in space and/or time are approximately independent: $p(D_{1},D_{2}|\zeta )\approx p(D_{1}|\zeta )p(D_{2}|\zeta )$.*

True independence holds, for example, when the clades corresponding to $D_{1},D_{2}$ are so distant that a reversible substitution process reaches stationarity on the edge connecting them. While we do not expect this to occur in real data, it seems like a reasonable approximation for studying distant subclades in a large, dense phylogeny which are evolving under a common evolutionary model. An example of the subsampling scheme we have in mind is when D= “all of the samples collected in Florida” (n≈81,000), $D_{1}=$ “all of the samples collected in Florida during June, 2020” ($b_{1}\approx 300$), and $D_{2}=$ “all of the samples collected in Florida during June, 2021” ($b_{2}\approx 5100$). Different subsampling schemes are possible depending on the data application, and these have an impact on the estimates; see supplementary Section S-3, Supplementary Material online for additional information.

Though incorrect, Heuristic 1 furnishes us with a useful formalism for performing large-scale inference, as we now demonstrate. Using the heuristic, we can approximate the posterior distribution (1) as

(2) $$p(\zeta ,T_{1\;:\;S}|D_{1\;:\;S})\propto \pi (\zeta )\prod_{i=1}^{S}p(D_{i}|T_{i})p(T_{i}|\zeta ),$$

where we used the array notation $T_{1\;:\;S}\equiv (T_{1},\ldots ,T_{S})$ to streamline the presentation.

Sampling from (2) is easier than sampling from the full posterior (1) since it decomposes into independent subproblems, and each subtree *$T_{i}$* is much smaller than the global phylogeny T. However, the normalizing constant in (2) remains intractable even for small trees, so naive sampling would still require expensive MCMC algorithms.

To work around this, we start by rewriting the last term in (2) as

$$p(T_{i}|\zeta )=p(T_{i}^{\mathrm{br}}|T_{i}^{\mathrm{topo}},\zeta )p(T_{i}^{\mathrm{topo}}|\zeta ).$$

As noted in the Introduction, the primary difficulty in Bayesian phylogenetic inference is navigating regions of topological tree space that have high posterior probability. If we could efficiently sample ${\hat{T}}_{i}^{\mathrm{topo}}∼p(T_{i}^{\mathrm{topo}}|\zeta )$, then the approximate posterior

(3) $$\hat{\;p}(\zeta ,T_{1\;:\;S}^{\mathrm{br}}|{\hat{T}}_{1\;:\;S}^{\mathrm{topo}},D_{1\;:\;S})\propto \pi (\zeta )\prod_{i=1}^{S}p(D_{i}|T_{i}^{\mathrm{br}},{\hat{T}}_{i}^{\mathrm{topo}})p(T_{i}^{\mathrm{br}}|{\hat{T}}_{i}^{\mathrm{topo}},\zeta )$$

would have the property that

(4) $$E_{{\hat{T}}_{1\;:\;S}^{\mathrm{topo}}}\hat{\;p}(\zeta ,T_{1\;:\;S}^{\mathrm{br}}|{\hat{T}}_{1\;:\;S}^{\mathrm{topo}},D_{1\;:\;S})=p(\zeta ,T_{1\;:\;S}^{\mathrm{br}}|D_{1\;:\;S}).$$

This leads to our second heuristic.

Heuristic 2. *Fitted tree topologies ${\hat{T}}_{1\;:\;S}^{\mathrm{topo}}$ obtained from subsets $D_{1},\ldots ,D_{m}$ pairwise satisfying Heuristic 1 are independent and approximately distributed as $p(T^{\mathrm{topo}}|\zeta )$.*

By “fitted trees” we mean trees estimated using any method, including fast heuristic algorithms such as UPGMA, or its extension to serially sampled time trees (sUPGMA; Drummond and Rodrigo 2000); maximum likelihood; or simply extracting subtrees from a high-quality reference phylogeny constructed by domain experts (e.g., Lanfear 2020). The heuristic can fail in various ways: in reality, tree reconstruction algorithms do not necessarily target the correct/any evolutionary prior, and there could be dependence between different trees if they are jointly estimated as part of a larger phylogeny. Also, our current implementation uses the data twice, once to estimate each tree, and again during model fitting to evaluate its phylogenetic likelihood. The tree inference procedure we used to analyze data in this paper is described more fully in the supplement (supplementary section S-2, Supplementary Material online). Note that we only utilize the *topological* information from these procedures; we still perform posterior inference over the branch lengths $T^{\mathrm{br}}$ as detailed below.

Setting these caveats aside, the point of Heuristic 2 is to endow our posterior estimates with some measure of phylogenetic uncertainty, without resorting to full-blown MCMC in tree space. By (4), the approximate likelihood (3) is unbiased for $p(\zeta ,T_{1\;:\;S}^{\mathrm{br}}|D_{1\;:\;S})$, and the latter quantity correctly accounts for phylogenetic variance in the posterior. However, since (3) conditions on ${\hat{T}}_{1\;:\;S}^{\mathrm{topo}}$, all of the remaining parameters to be sampled are continuous, and the problem becomes much easier.

We stress that our method is not capable generating useful samples from the posterior distribution p(T∣D), that is of the overall transmission tree given the original dataset D. But, as noted above, in skyline-type models the main object of interest is the evolutionary posterior p(ζ∣D). In Section “Results,” we demonstrate that the heuristic, subsampling-based approach developed here yields a fairly sharp posterior on ζ, although still utilizing a large amount of information from D.

#### Stochastic Variational Inference

Since (3) is a distribution over continuous, real-valued parameters, it is amenable to variational inference (Jordan et al. 1999). As noted in the introduction, variational Bayesian phylogenetic inference has previously been studied by Zhang and Matsen (2019), Zhang (2020) and Fourment and Darling (2019). Our approach is most related to the latter since we do not optimize over the topological parameters of our model in any way. Because we are operating in a different data regime than either of these two pre-pandemic papers, we further incorporated recent advances in large-scale Bayesian inference in order to improve the performance of our method.

Given a Bayesian inference problem consisting of data x and model parameters z, traditional VI seeks to minimize the Kullback–Leibler (KL) divergence between the true posterior of interest and family of tractable approximating distributions Q:

q*(z)=argminq(z)∈QKL(q(z)∥p(z∣x)).

We cannot carry out this minimization as the KL divergence still requires evaluating the intractable quantity p(x). However,

(5) $$\begin{array}{rl} \text{KL}(q(z)∥p(z|x)) & =E(\log\;q(z))-E(\log\;p(z|x))\\ & =E(\log\;q(z))-E(\log\;p(x,z))+\log\;p(x)\\ & =-\text{ELBO}(q(z))+\text{const.} \end{array}$$

where the expectations are with respect to the variational distribution q, and

(6) $$\text{ELBO}(q(z))\;:\;=E_{z∼q(z)}[\log\;p(x,z)-\log\;q(z)]$$

is known as the evidence lower bound. Hence, minimizing the divergence between the true and variational posterior distributions is equivalent to maximizing the ELBO.

For VI involving complex (non-exponential family) likelihoods, the ELBO is generally approximated by replacing the first term in (6) by a Monte Carlo estimate:

(7) $$E_{z∼q(z)}\log\;p(x,z)\approx \frac{1}{B}\sum_{i=1}^{B}\log\;p(x,z_{i});\;z_{1},\ldots ,z_{B}∼q(z)\text{ i.i.d.}$$

where B=1 is a common choice. Each evaluation of the complete likelihood logp(x,z) requires a full pass over the data, which can be prohibitive when the data are large. Stochastic variational inference (SVI; Hoffman et al. 2013) addresses this problem through stochastic optimization. Many Bayesian models naturally factorize into a set of shared, global hidden variables, and sets of local hidden variables which are specific to each observation. Each observation is conditionally independent of all others given its local parameters. Hoffman et al. show how models of this form are well suited to stochastic gradient descent. Specifically, they derive an unbiased gradient estimator of the ELBO (6) which operates on a single, randomly sampled data point at each iteration. The algorithm tends to make better progress in early stages when the variational approximation to the shared global parameters is still quite inaccurate (Hoffman et al. 2013).

By design, the model we derived above is suited to SVI. In equation (3), the evolutionary parameters ζ are shared among all datasets, whereas the branch length parameters $T_{i}^{\mathrm{br}}$ are specific to the ith dataset $D_{i}$. We therefore refer to ζ as the global parameter, and the vectors of dataset-specific branch lengths $T_{1\;:\;S}^{\mathrm{br}}$ as local parameters. Our algorithm proceeds by iteratively sampling a single dataset $D_{i}$ and taking a noisy (but unbiased) gradient step. Note that, because our model is not in the exponential family, we cannot employ the elegant coordinate-ascent scheme originally derived by Hoffman et al. Instead, we numerically optimize the ELBO using differentiable programming (see below).

#### Model Parameterization

It remains to specify our model parameterization and the class of distributions Q that are used to approximate the posterior. Recall from section “Notation and Model” that the global parameter ζ includes the effective reproduction number R(t), rate of becoming uninfectious δ(t), and sampling fraction s(t). We follow earlier work (Gill et al. 2013) in assuming that these rate functions are piecewise constant over time, with changepoints whose location and number are fixed *a priori*. The changepoints are denoted $t=(t_{1},\ldots ,t_{m})$ satisfying $0=t_{0}<t_{1}<\cdots <t_{m}<t_{m+1}=\infty$. Thus,

$$R(t)=\sum_{i=1}^{m+1}R_{i}1_{\left\{t\in [t_{i-1},t_{i})\right\}}(t),$$

where the transmission rates in each time interval are denoted $R=(R_{1},\ldots ,R_{m})\in R_{>0}^{m}$. The rate of becoming uninfectious and sampling fraction are similarly denoted by $\delta \in R_{>0}^{m}$ and $s\in [0,1{]}^{m}$, respectively. Finally, a Gaussian Markov random field (GMRF) smoothing prior is used to penalize consecutive differences in the log rates (Minin et al. 2008). To account for the fact that each rate parameter may have varying degrees of smoothness and also could be on different scales, each rate parameter has a corresponding precision hyperparameter $\tau_{R},\tau_{\delta }$, and $\tau_{s}$.

An extension of the BDSKY model allows for additional sampling efforts at each time $t_{k}$. Infected individuals are sampled with probability $\rho_{k}$ at time $t_{k}$. When all sequences are sampled serially without the added sampling effort, $\rho_{k}=0$ for 1≤k≤m. When all sequences are sampled contemporaneously, ψ=0, $\rho_{k}=0$ for 1≤k≤m−1, and $\rho_{m}>0$. For our work, we only consider cases where $\rho_{k}=0$ for 1≤k≤m−1. We define $b_{s}$ as the number of sequences sampled serially, and $b_{m}$ to be the number of sequences sampled at time $t_{m}$. In other words, $b_{m}$ is the number of contemporaneously sampled sequences at time $t_{m}$. Note that $b=b_{m}+b_{s}$. The sample times of the $b_{s}$ serially sampled sequences are denoted by ${\tilde{y}}^{\left(i\right)}=(y_{1}^{\left(i\right)},\ldots ,y_{b_{s}}^{\left(i\right)})$. Because the sequences sampled at $t_{m}$ have the largest sample time, ${\tilde{y}}^{\left(i\right)}$ is just a truncated version of $y^{\left(i\right)}$. When all sequences are sampled serially, $y^{\left(i\right)}={\tilde{y}}^{\left(i\right)}$. To conserve notation, from this point onward, we will use $y^{\left(i\right)}$ to refer to ${\tilde{y}}^{\left(i\right)}$.

The final remaining global parameter is the epidemic origin time $t_{0}$. In order for the model to be well defined, this must occur earlier than the earliest sampling time in any of the S subsamples. Therefore, we set $t_{0}+x_{1}=y_{\min}$, where $y_{\min}$ is the earliest sampling time across all subsamples, and place a prior on $x_{1}>0$ as detailed below.

Given the sampling times and estimated tree topology ${\hat{T}}_{i}^{\mathrm{topo}}$, we can identify each local parameter $T_{i}^{\mathrm{br}}$ with a vector $h^{\left(i\right)}\in R_{>0}^{b-1}$ giving the height of each internal node when enumerated in preorder. Hence the height of the root node is $h_{1}^{\left(i\right)}$. We follow the parameterizations set forth by Fourment and Darling (2019). In order for a sampled tree to be valid, we must have $h_{j}^{\left(i\right)}<h_{\mathrm{pa}(j)}^{\left(i\right)}$ for every j. Here pa(j) denotes the parent node of node j. This constraint can be met by setting the height of internal node j as $h_{\;j}^{\left(i\right)}=p_{\;j}^{\left(i\right)}(h_{\mathrm{pa}(j)}^{\left(i\right)}-h_{d(j)}^{\left(i\right)})$ where d(j) is the earliest sampled tip from the set of descendants of j and $p_{j}^{\left(i\right)}\in [0,1]$. Finally, let $x_{1}^{\left(i\right)}$ denote the distance of the root node from the origin measured forward in time. We must have $t_{0}<x_{1}^{\left(i\right)}<y_{1}^{\left(i\right)}$ since the root node of $T_{i}$ has to be between the origin and the earliest sample time. Therefore we set $x_{1}^{\left(i\right)}-t_{0}=r^{\left(i\right)}y_{1}^{\left(i\right)}$ for some $r^{\left(i\right)}\in [0,1]$, and calculate the root height $h_{1}^{\left(i\right)}$ from it. Under this parameterization, the set of local variables $z^{\left(i\right)}=(p_{1}^{\left(i\right)},\ldots ,p_{b-1}^{\left(i\right)},r^{\left(i\right)})\in [0,1{]}^{b}$ is a set of proportions, with transformations to switch between parameterizations for BDSKY and the observed data likelihood.

#### Variational Approximating Family

We make a standard mean-field assumption, which posits that members of Q completely factorize into a product of independent marginals. Letting $\zeta =(R_{1},\ldots ,R_{m},\delta_{1},\ldots ,\delta_{m},s_{1},\ldots ,s_{m})$ denote the collection of all global parameters defined above, and recalling the definition of $z^{\left(i\right)}$ in the preceding paragraph, we assume that

(8) $$q(\zeta ,z^{\left(1\right)},\ldots ,z^{\left(m\right)})=\prod_{i}q(\zeta_{i}|\pi_{i})\prod_{\;j}\prod_{k}q(z_{\;j}^{\left(k\right)}|\phi_{\;j}^{\left(k\right)}),$$

where we have introduced variational parameters $\pi_{i}$ and $\phi_{\;j}^{\left(k\right)}$ corresponding to each marginal distribution. The distributions $q(\zeta_{i}|\pi_{i})$ and $q(z_{\;j}^{\left(k\right)}|\phi_{\;j}^{\left(k\right)})$ are (suitably transformed) Gaussians, so that $\pi_{i},\phi_{\;j}^{\left(k\right)}\in R\times R_{\geq 0}$ each comprises a real location parameter and non-negative scale parameter. In our model, all latent parameters, local or global, are constrained to be positive (e.g., R,δ) or in the unit interval (e.g., s, $z^{\left(i\right)}$). For each parameter, we take q to be an appropriately transformed normal distribution. For positive parameters, we use an exponential transformation, and for parameters constrained to be in (0,1) we use an expit (inverse logistic) transformation.

#### Implementation using Differentiable Programming

Our Python software implementation uses automatic differentiation in order to efficiently optimize the variational objective function (Kucukelbir et al. 2017; Bradbury et al. 2018). We sample from the variational distribution and estimate the gradient of the (7) objective function with respect to the variational parameters π and ϕ using Monte Carlo integration (cf. eq. 7). Gradients of the phylogenetic likelihood are computed in linear time using the recent algorithm of Ji et al. (2020). The complete fitting algorithm is shown in Algorithm 1.

**Algorithm 1:** Variational Bayesian Skyline (VBSKY)

**Input:** Data set D, sampling times y; Fixed parameters m,ν,S,b; Step size α.
**for** * i=1→S * **do**
Sample with replacement b times from the data to get subsample $D_{i},y^{\left(i\right)}$.
Estimate the tree topology ${\hat{T}}_{i}^{topo}$.
**end**
Initialize π,ϕ randomly.
**while** not converged **do**
**for***i=1→B***do**
Draw M samples $z^{\left(i\right)}∼q(\cdot |\phi^{\left(i\right)}),$ζ∼q(⋅∣π).
Approximate $\nabla_{\phi^{\left(i\right)}}L$ and $\nabla_{\pi }L$ using MC integration.
Update $\phi^{\left(i\right)}\leftarrow \phi^{\left(i\right)}+\alpha \nabla_{\phi^{\left(i\right)}}L.$
Update $\pi \leftarrow \pi +\alpha \nabla_{\pi }L.$
**end**
**end**
**Return** * π,ϕ *

## Supplementary Material

msac154_Supplementary_Data

## Data Availability

All of the data analyzed in this manuscript are publicly available. A Python implementation of our method, as well as Jupyter notebooks which reproduce our results, are located at https://github.com/jthlab/vbsky.
