# Supplementary material for: Variational Phylodynamic Inference Using Pandemic-scale Data
Source: Mol Biol Evol. 2022 Jul 11;39(8):msac154. doi: 10.1093/molbev/msac154 (PMC9348775; doi:10.1093/molbev/msac154)
Supplement: msac154_Supplementary_Data [file msac154_supplementary_data.zip › supp.pdf]

Supplemental Materials

S-1 Birth Death Skyline

In this section we review the birth-death skyline (BDSKY) model of Stadler et al. (2013). BDSKY is a forward time model which begins with a single individual at time t_0 and ends at t_m. Throughout this section, we will refer to the start of the process as the origin. As with other skyline methods, the parameters of the model are allowed to vary over time. Specifically, given a vector $\mathbf{t} = (t_0, t_1, \ldots, t_m)$ satisfying $0 < t_1 < \cdots < t_m$, parameters are fixed between each t_k and t_{k-1}, and allowed to vary m times. The transmission rates are denoted by the vector $\boldsymbol{\lambda} \in \mathbb{R}^m_{>0}$. Similarly, the death rates are given by the vector $\boldsymbol{\mu}$ and the sampling rates by the vector $\boldsymbol{\psi}$ where each $\mu_k > 0$ and $\psi_k > 0$. In the interval $[t_{k-1}, t_k)$, every infected individual transmits at rate λ_k, recovers at rate μ_i, and is sampled at rate ψ_k. For ease of notation, we denote $\lambda(t), \mu(t)$, and $\psi(t)$ as the transmission rate, uninfectious rate, and sampling rate at time t. We assume that after sampling, the individual can no longer transmit. This assumption holds in reality for many viruses as sampling is often followed by treatment or changes in behavior that would curb or limit spread. For example, those sampled with HIV would undergo antiretroviral therapy or those sampled with COVID-19 would quarantine themselves.

As described in the main text, the BDSKY model also allows for additional sampling efforts at each time t_k. For the reader's convenience we reproduce the notation here. All infected are sampled with rate ρ_k at time t_k. When all sequences are sampled serially without the added sampling effort, $\rho_k = 0$ for $1 \le k \le m$. When all sequences are sampled contemporaneously, $\boldsymbol{\psi} = \mathbf{0}$, $\rho_k = 0$ for $1 \le k \le m-1$, and $\rho_m > 0$. For our work, we only consider cases where $\rho_k = 0$ for $1 \le k \le m-1$. We define b_s as the number of sequences sampled serially, and b_m to be the number of sequences sampled at time t_m. In other words, b_m is the number of contemporaneously sampled sequences at time t_m. Note that $b = b_m + b_s$. The sample times of the b_s serially sampled sequences are denoted by $\tilde{\boldsymbol{y}}^{(i)} = (y_1^{(i)}, \ldots, y_{b_s}^{(i)})$. Because the sequences sampled at t_m have the largest sample time, $\tilde{\boldsymbol{y}}^{(i)}$ is just a truncated version of $\boldsymbol{y}^{(i)}$. When all sequences are sampled serially, $\boldsymbol{y}^{(i)} = \tilde{\boldsymbol{y}}^{(i)}$. To conserve notation, from this point onward, we will use $\boldsymbol{y}^{(i)}$ to refer to $\tilde{\boldsymbol{y}}^{(i)}$. The $b-1$ transmission event times are denoted by $\boldsymbol{x}^{(i)} = (x_1^{(i)}, \ldots, x_{b-1}^{(i)})$ where $0 < x_1^{(i)} < \cdots < x_{b-1}^{(i)}$.

The number of lineages that began before t_k and are extant at t_k is n_k. Any tree $\mathcal{T}_i$ induced by the BDSKY model is described by its tree topology $\mathcal{T}_i^{\text{topo}}$, the transmission times $\boldsymbol{x}^{(i)}$, and the sampling times $\boldsymbol{y}^{(i)}$. Letting S be the event that at we observe at least one sample, the probability density of a tree under the BDSKY model is

$$p(\mathcal{T}_i \mid \boldsymbol{\lambda}, \boldsymbol{\mu}, \boldsymbol{\psi}, \boldsymbol{\rho}, \boldsymbol{t}, S) = \frac{q_1(0)\rho_m^{b_m}}{1 - p_1(0)} \prod_{k=1}^{b-1} \lambda_{I(x_k^{(i)})} q_{I(x_k^{(i)})}(x_k^{(i)}) \prod_{k=1}^{n_s} \frac{\psi_{I(y_k^{(i)})}}{q_{I(y_k^{(i)})}(y_k^{(i)})} \prod_{k=1}^{m} q_{k+1}(t_k)^{n_k}, \tag{S1}$$

where $I(t) = k$ if $t_{k-1} \le t < t_k$, and for $k = 1, \ldots, m$ and $t_{k-1} \le t < t_k$,

$$\begin{aligned}
A_k &= \sqrt{(\lambda_k - \mu_k - \psi_k)^2 + 4\lambda_k\psi_k} \\
B_k &= \frac{(1 - 2(1-\rho_k)p_{k+1}(t_i))\lambda_k + \mu_k + \psi_k}{A_k} \\
p_k(t) &= \frac{\lambda_k + \mu_k + \psi_k - A_k \frac{e^{A_k(t_k - t)}(1+B_k) - (1-B_k)}{e^{A_k(t_k - t)}(1+B_k) + (1-B_k)}}{2\lambda_k} \\
q_k(t) &= \frac{4e^{-A_k(t-t_k)}}{(e^{-A_k(t-t_k)}(1+B_k) + (1-B_k))^2},
\end{aligned}$$

and $p_{m+1}(t_m) = 1$.

S-2 Estimating the tree topology

In this section we explain how we estimate the tree topology $\hat{\mathcal{T}}_i^{\text{topo}}$ for each subsample $\mathcal{D}_i$. We employed a simple heuristic method by fitting serial-sample unweighted pair grouping method with arithmetic means (sUPGMA) (Drummond and Rodrigo, 2000). As the name alludes to, sUPGMA is a tree reconstruction algorithm based on the unweighted paired group method with arithmetic means (UPGMA) (Sneath and Sokal, 1973).

We first describe the UPGMA algorithm followed by the sUPGMA algorithm. Both algorithms require a pairwise distance matrix. Taking the sequences of our subsample, $\mathcal{D}_i \in \{A, C, G, T\}^{b\times L}$, we simply take the Hamming distance between all $\binom{b}{2}$ pairs of sequences. That is for a given pair of sequences $s, t \in \mathcal{D}_i$, the distance is $d(s,t) = \sum_{k=1}^{L} \mathbf{1}_{(s_k \neq t_k)}$. For clusters, the mean distance between each element in the clusters. That is

$$d(A, B) = \frac{1}{|A||B|} \sum_{s\in A} \sum_{t \in B} d(s,t),$$

where A and B both represent clusters. At each step the two clusters with the smallest distance between them are combined into a new cluster, and the distances are recalculated between the newly formed cluster and the other clusters. Clusters can be made up of a single sequence. We start with b clusters initially and reduce the number of clusters by one at each step. This is repeated until there is only one single cluster.

While we could naively use UPGMA to get our tree topologies, because UPGMA does not account for sample times, it is possible the algorithm would give us topologies that are impossible given the sample times. We use sUPGMA to ensure that the estimated topology is not only possible, but realistic. Consider our subsample of sample times $\boldsymbol{y}^{(i)} = \{y_1^{(i)}, y_2^{(i)}, \ldots, y_b^{(i)}\}$. Let $d(u_i, v_j)$ be the distance between ith sequence with sample time $y_u^{(i)}$ and the jth sequence with sample time y_v. We assume that $u < v$. and model $d(u_i, v_j)$ by its expectation, $\mathbb{E}(d(u_i, v_j)) = \Theta_u + \omega(y_v^{(i)} - y_u^{(i)})$, where Θ_u is the expected average distance between any two sequences at time $y_u^{(i)}$, and ω is the expected number of substitutions per unit time. The procedure for sUPGMA is as follows.

1. Estimate the set of parameters $\{\Theta_1, \ldots, \Theta_q, \omega\}$ using regression:

   $$d(u_i, v_j) = \sum_{k=1}^{q} \Theta_k X_k + \omega(y_v^{(i)} - y_u^{(i)}) + \epsilon,$$

   where $X_k = 1$ if $k = u$ and $k = 0$ otherwise.

2. Correct the original pairwise distances

   $$c(u_i, v_j) = d(u_i, v_j) + \omega(y_u^{(i)} + y_v^{(i)} - 2y_1^{(i)}).$$

3. Cluster using the UPGMA algorithm as described earlier.

We note that while the complete sUPGMA algorithm returns both the tree topology and the branch lengths, we only use this procedure to obtain the tree topology. As branch lengths are continuous variables, we will estimate those using stochastic variational inference. Although there are maximum likelihood based tree reconstruction methods we could use such as IQ-TREE (Minh et al., 2020), since we are only concerned with the topology rather than the entire tree, we prioritize the faster algorithm.

S-3 Subsampling strategies

The algorithm described in the main text generates an ensemble of trees by randomly subsampling the data. We explored several possible ways of generating these samples:

- **Simple random sampling**. The simplest way to generate these is to sample lineages uniformly at random without replacement. This approach is most in line with the heuristics which were used to motivate our approach, since successive random samples are more likely to be approximately independent in a large infection tree. However, it may be suboptimal if the sampling of infected nodes tends to be biased towards a particular point in time and/or space.
- **Biased sampling**. In many situations there is a noticeable bias in the date or location of sampled infections (e.g. Figure S9). For example, if most of the samples were collected in the recent past, most of the trees in the ensemble will have tips from near the present, making it difficult to estimate transmission events further back in time. To counteract this effect, we also explored a "biased" sampling scheme which samples lineage uniformly with respect to time. We split the data by the quarter in which the sequence was sampled, where the first quarter of each year was defined to be the first three months (January, February, March) of the year, and so on. Then, instead of randomly sampling to generate the ensemble of trees, for each tree the tips were restricted to only one quarter. We also enforced the number of trees per quarter to be approximately equal. One caveat is that this stratified sampling approach could bias the estimates of the sampling rate.
- **Clustered sampling**. A third possibility, suggested by a referee, is to sample the tips of small subclades in a pre-estimated infection tree. To the extent that the tree inference procedure is likely to produce tree estimates which are temporally and spatially coherent, this should have a similar effect as the biased sampling procedure described above.

S-4 Hyperparameter Tuning

Apart from the algorithmic choices described in the preceding sections, VBSKY has two main tuning parameters that can be adjusted: the number of tips in each subsample (denoted b in the main sect), and the number of subsamples of the overall dataset $\mathcal{D}$ (denoted S in the preceding section). Increasing either enables us to analyze more sequences, but at the expense of additional computation time.

To understand the effect of the number of trees, we examined the posterior of the effective reproductive number and the sampling rate of the UK and the USA while fixing the number of tips and varying the number of trees. We set the number of tips to be 200 and examined the posterior for each number of trees in the set $\{10, 25, 50, 100, 150\}$. Patients with mild bouts of COVID-19 are generally not infectious after 10 days of symptom onset (Arons et al., 2020; Bullard et al., 2020). The rate of becoming uninfectious is the inverse of the number of infectious days. As one unit of time corresponds to one year, the estimated value for δ is given by $1/10 \times 365 = 36.5$. Using this, we fixed the uninfectious rate to be 36.5 to avoid nonidentifiability issues since we cannot estimate R, δ, and s simultaneously (Stadler, 2009; Louca and Pennell, 2020). For the GMRF smoothing prior, we chose a relatively uninformative hyperprior distribution with large variance for the parameters of the smoothing prior. In particular, we selected a gamma distribution distribution with parameters $a = b = 0.001$, giving a mean of 1 and variance of 1000. As a rough estimate of the sampling rate, we also chose the prior for s to be a $\text{Beta}(0.02, 0.98)$ distribution with expectation 0.02, as the ratio of sampled sequences to the number of cumulative cases is around 0.02. The remaining priors are shown in the first line of Table **??**.

Figure S22 shows the posterior of R for both the UK and the USA when varying the number of trees. Figure S23 shows the posterior for s. The figures indicate a larger difference when the number of trees is 10 compared to any greater number of trees. The median and credible interval for R was much smaller and the median and credible interval for s was much larger closer to the present when the number of trees was 10. The credible intervals when the number of trees was 10 was also much wider. A closer inspection showed that this also seems to be the case when the number of trees is 25, albeit to a smaller degree. When we increased the number of trees to 50, this difference mostly disappeared.

We performed a similar study to understand the effect of varying the number of tips. We fixed the number of trees as 50 and adjust the number of tips to values in the set $\{50, 100, 200, 400\}$, and examined the posteriors of R and s while holding δ fixed. Similar to above, varying the number of tips does not appear

to have a large effect on the results. Using only 50 tips per tree resulted in a wider credible interval for the UK and the USA for both R and s. Figure S24 shows that using 50 tips also leads to flatter estimates for R further back in the past. This is likely the result of trees with fewer tips having fewer transmission events further back in the past which can be used to estimate R.

Overall, regardless of the number of tips or trees used, the posterior estimates of both R and s for both the UK and the USA are similar. However, increasing the number of trees decreases the variances in posterior estimates of R and s, and also results in more accurate estimates of both parameters towards the present. This improvement seems to plateau after increasing the number of trees to 50. Similarly, increasing the number of tips can increase the power to detect changes in R and s further back in the past, but using too many tips can lead to more erratic estimates of the parameters towards the present.

Keeping this in mind while also noting that increasing the number of trees and tips can incur large computational costs, using 50 trees with 200 tips leads to sharper estimates of the posterior without requiring excessive computation. After trying various values for the step size and batch size for our stochastic variational inference procedure, we chose the values of those hyperparameters as 1 and 1.

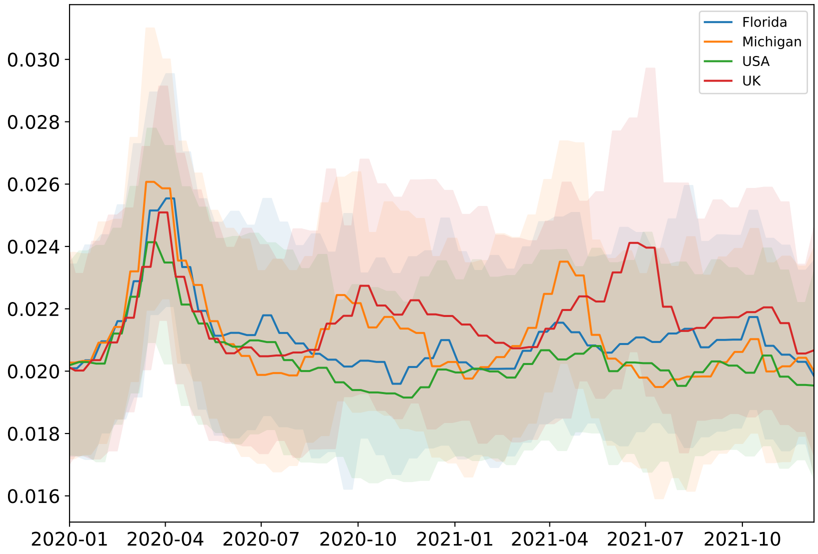

Figure S1: The posterior median and equal-tailed 95% credible interval of s for all regions using biased sampling.

When comparing the posteriors when the number of tips is 100 or 200, only minor differences appeared. Using 200 tips did seem to lead to better detection of changes in R and s further back in the past. Looking at Figure S24, using 400 tips per tree led to a sharper decrease in R towards the present. Figure S25 shows that using 400 tips generally led to slightly larger estimates of s at all points in time.

References

Melissa M Arons, Kelly M Hatfield, Sujan C Reddy, Anne Kimball, Allison James, Jesica R Jacobs, Joanne Taylor, Kevin Spicer, Ana C Bardossy, Lisa P Oakley, et al. Presymptomatic SARS-CoV-2 infections and transmission in a skilled nursing facility. *New England journal of medicine*, 382(22):2081–2090, 2020.

Jared Bullard, Kerry Dust, Duane Funk, James E Strong, David Alexander, Lauren Garnett, Carl Boodman, Alexander Bello, Adam Hedley, Zachary Schiffman, et al. Predicting infectious severe acute respiratory syndrome coronavirus 2 from diagnostic samples. *Clinical Infectious Diseases*, 71(10):2663–2666, 2020.

Alexei Drummond and Allen G Rodrigo. Reconstructing genealogies of serial samples under the assumption of a molecular clock using serial-sample upgma. *Molecular Biology and Evolution*, 17(12):1807–1815, 2000.

Stilianos Louca and Matthew W Pennell. Extant timetrees are consistent with a myriad of diversification histories. *Nature*, 580(7804):502–505, April 2020. ISSN 0028-0836, 1476-4687. doi: 10.1038/s41586-020-2176-1.

Bui Quang Minh, Heiko A Schmidt, Olga Chernomor, Dominik Schrempf, Michael D Woodhams, Arndt Von Haeseler, and Robert Lanfear. Iq-tree 2: New models and efficient methods for phylogenetic inference in the genomic era. *Molecular biology and evolution*, 37(5):1530–1534, 2020.

Peter HA Sneath and Robert R Sokal. *Numerical taxonomy. The principles and practice of numerical classification.* 1973.

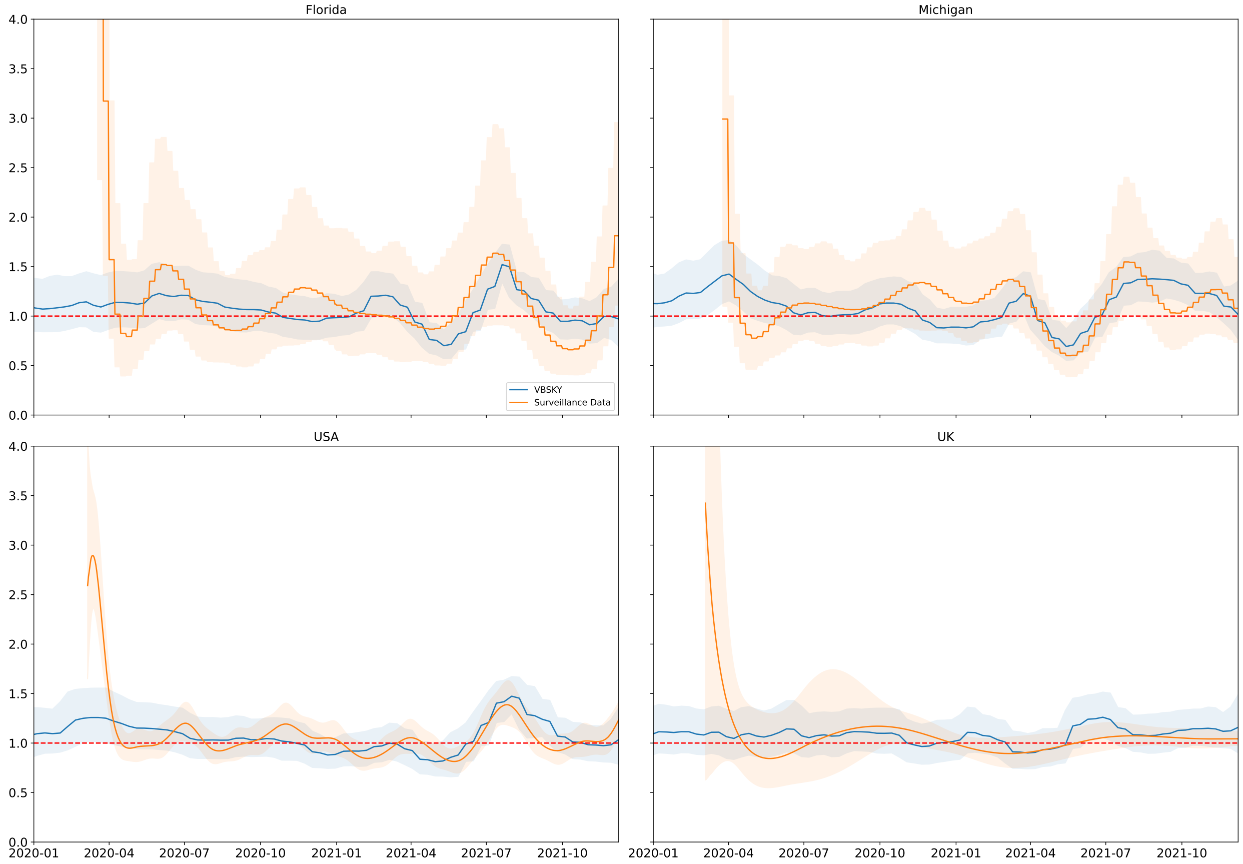

Figure S2: Posterior for R for using an uninformative smoothing prior. VBSKY estimates are in blue. The orange estimates are derived from surveillance data. For each method the posterior median and equal-tailed 95% credible interval are shown. The dotted red line is $R = 1$.

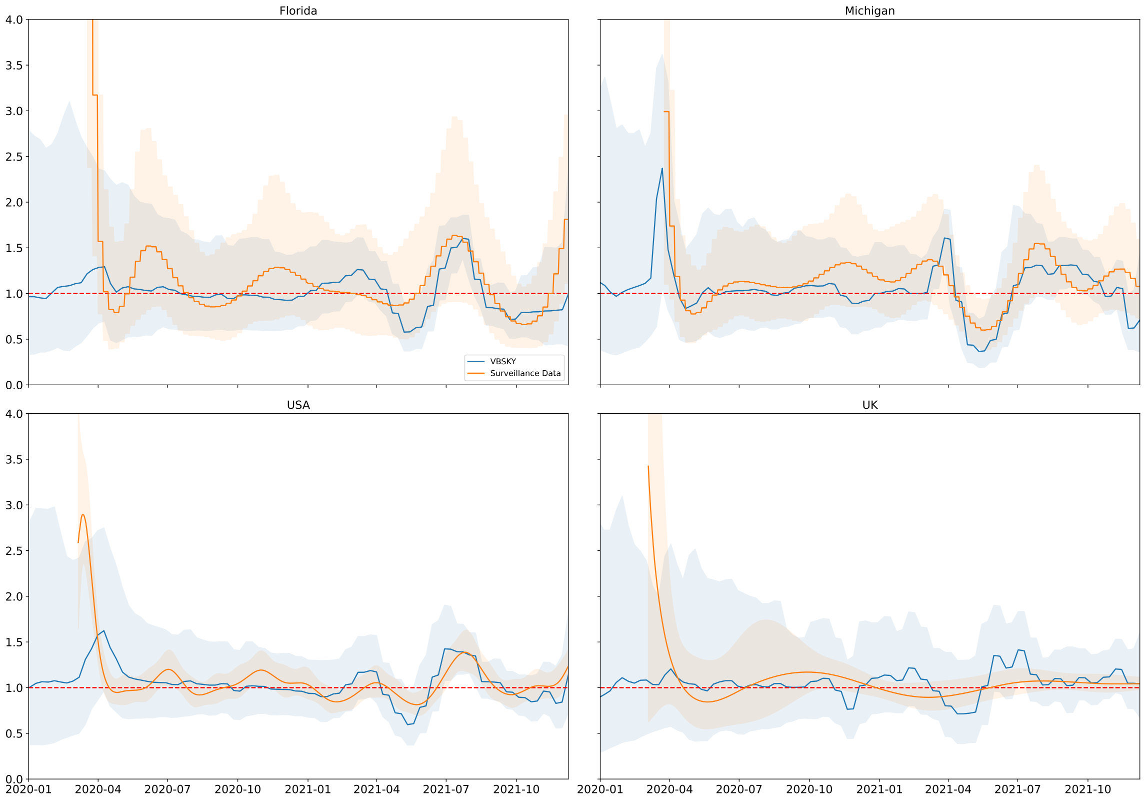

Figure S3: Posterior for R using less smoothing. VBSKY estimates are in blue. The orange estimates are derived from surveillance data. For each method the posterior median and equal-tailed 95% credible interval are shown. The dotted red line is $R = 1$.

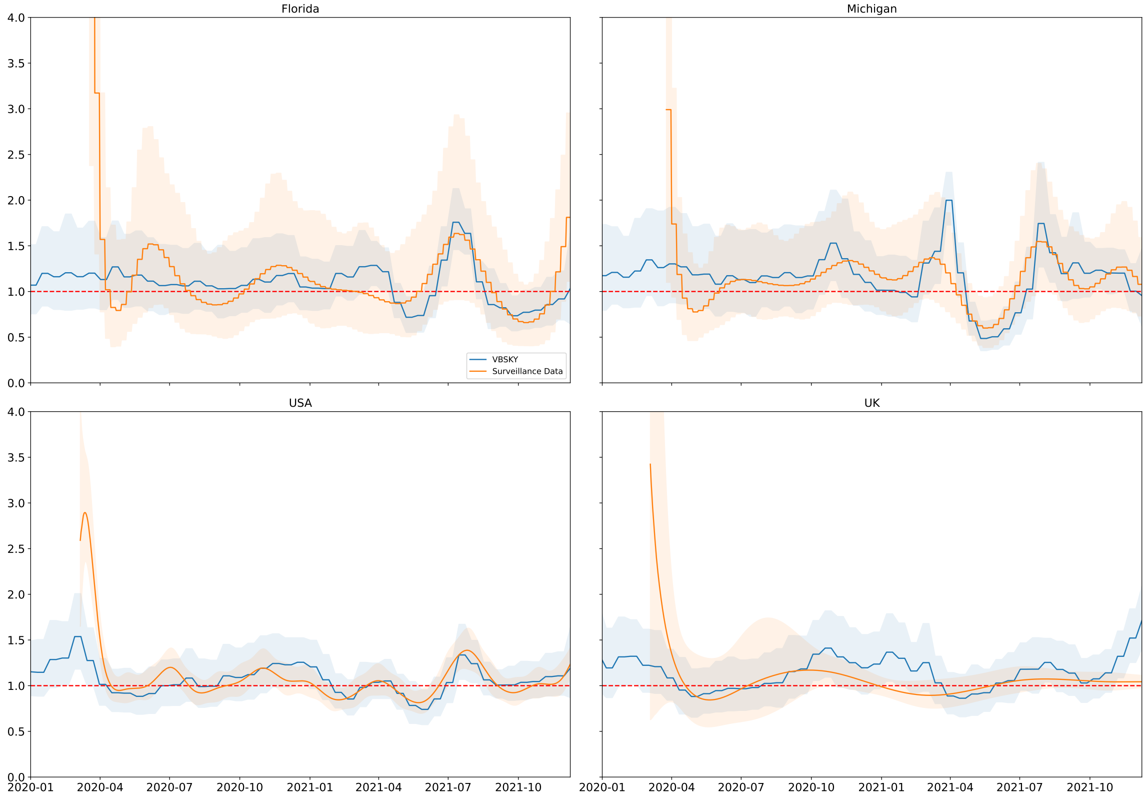

Figure S4: Posterior for R using clustered sampling. VBSKY estimates are in blue. The orange estimates are derived from surveillance data. For each method the posterior median and equal-tailed 95% credible interval are shown. The dotted red line is $R = 1$.

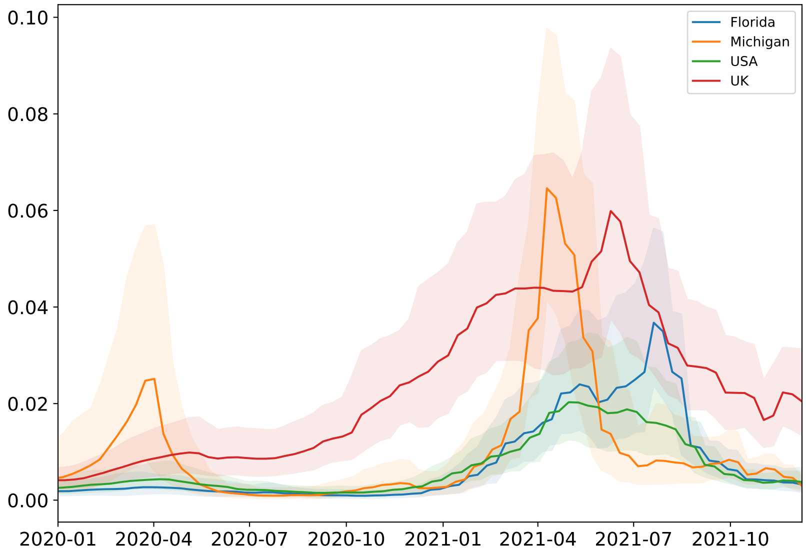

Figure S5: The posterior median and equal-tailed 95% credible interval for s for all regions using an uninformative smoothing prior.

Tanja Stadler. On incomplete sampling under birth–death models and connections to the sampling-based coalescent. *Journal of theoretical biology*, 261(1):58–66, 2009.

Tanja Stadler, Denise Kühnert, Sebastian Bonhoeffer, and Alexei J Drummond. Birth–death skyline plot reveals temporal changes of epidemic spread in HIV and hepatitis C virus (HCV). *Proceedings of the National Academy of Sciences*, 110(1):228–233, 2013.

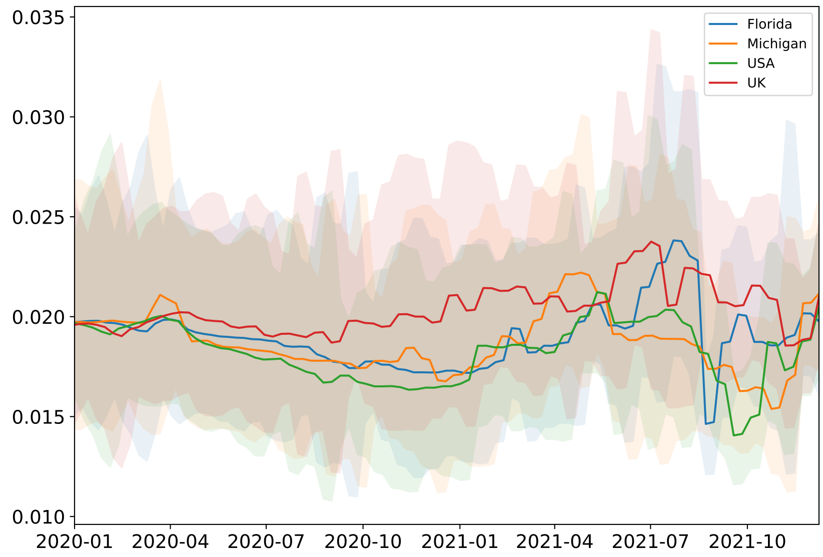

Figure S6: The posterior median and equal-tailed 95% credible interval for s for all regions using less smoothing.

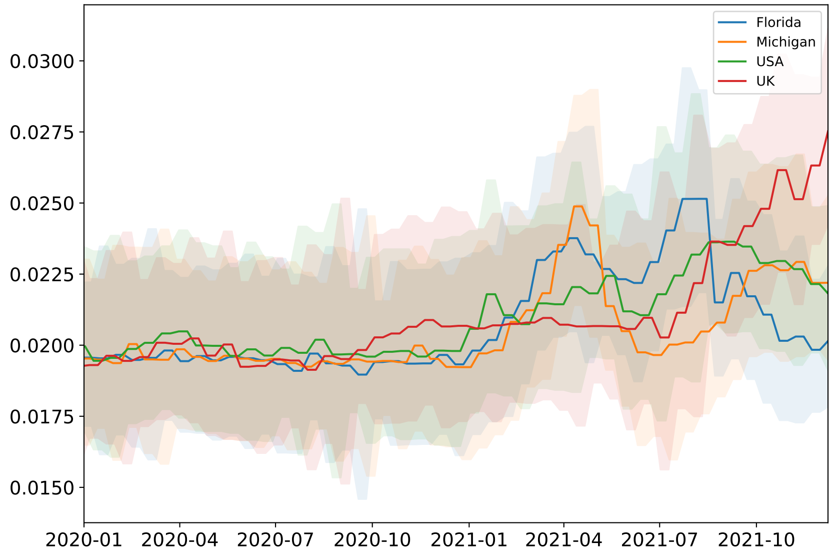

Figure S7: The posterior median and equal-tailed 95% credible interval for s for all regions using clustered sampling.

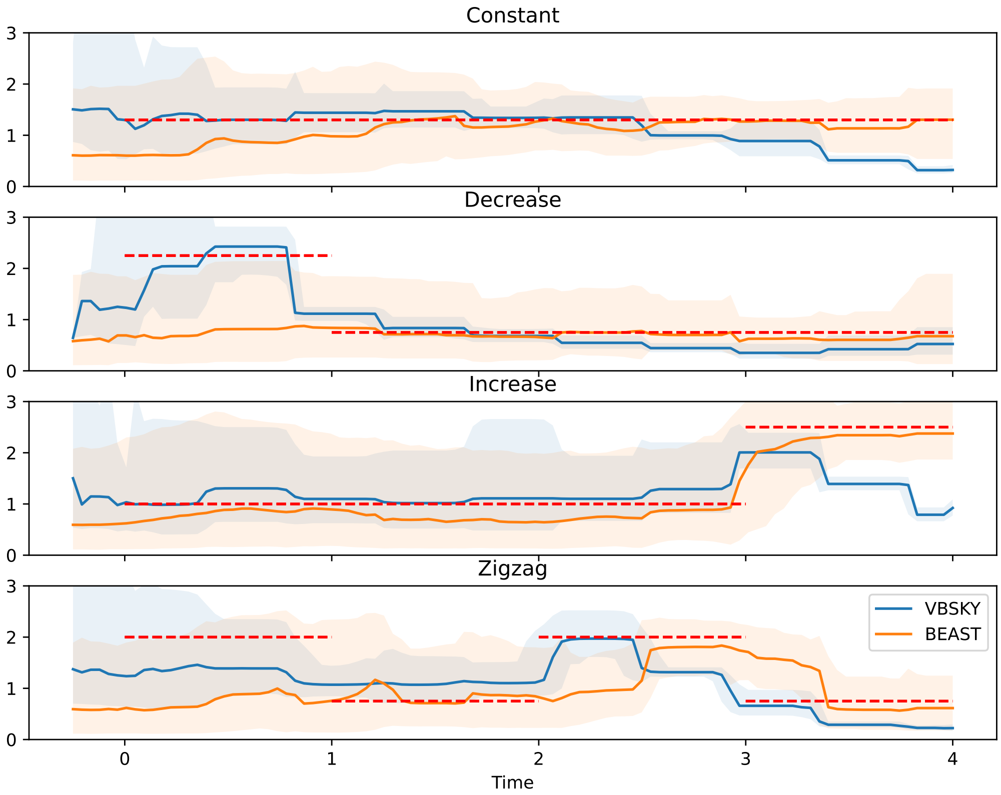

Figure S8: Median of the medians and the equal-tailed 95% credible intervals of the posteriors of the effective reproductive number over time of the 10 simulations for each scenario using VBSKY on the smaller data sets used for the BEAST analysis. The dotted red line is the true effective reproductive number over time.

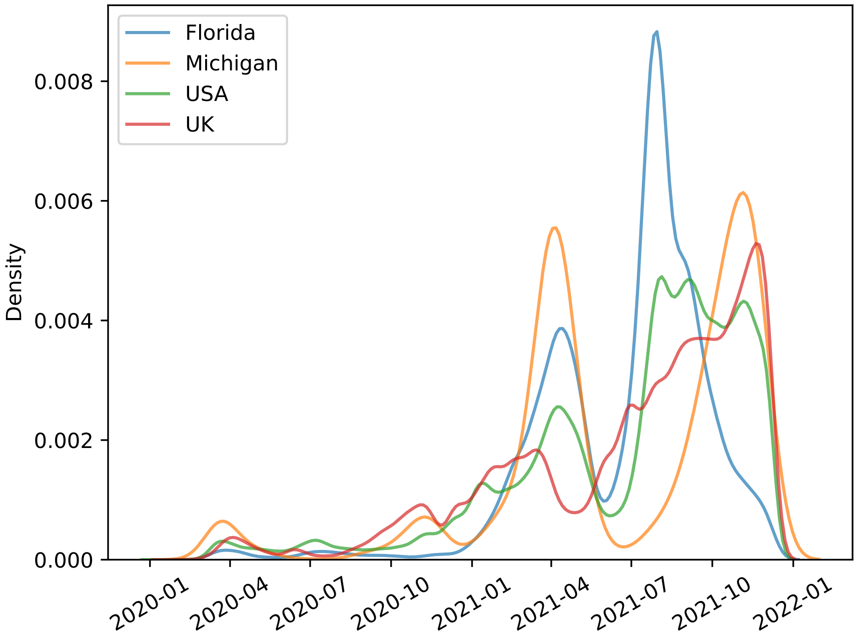

Figure S9: Density estimate of sample times.

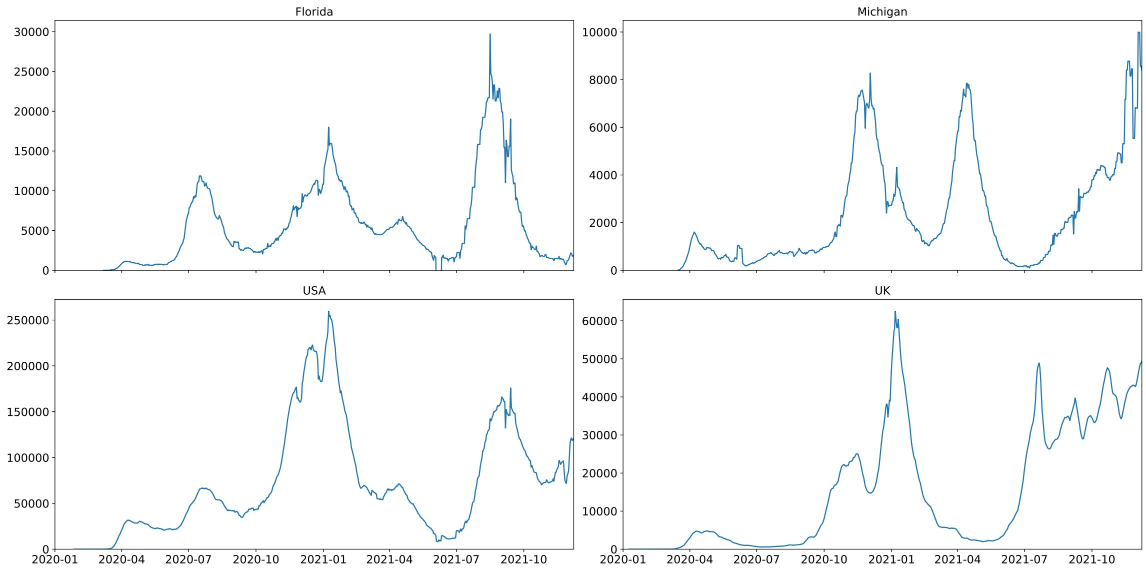

Figure S10: Daily new cases of COVID-19 over time.

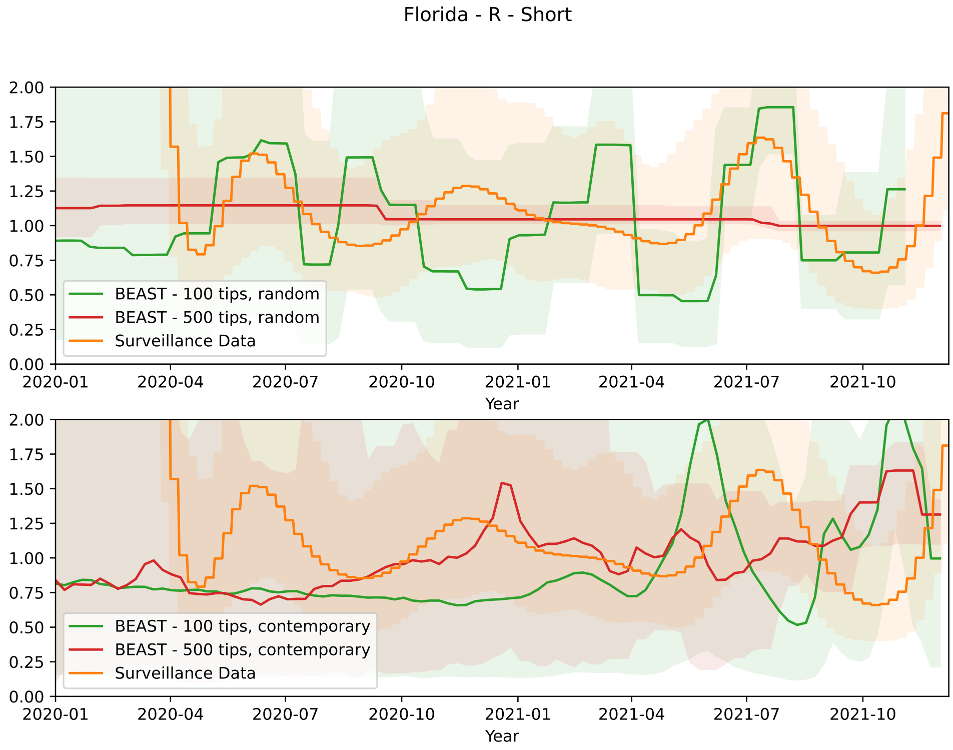

Figure S11: The posterior median and equal-tailed 95% credible interval of R for Florida given by BEAST. The top panel contains randomly sampled data, while the bottom contains the most recent available samples. The sampler was allowed to run as long as it VBSKY to analyze the Florida data. This is referred to as the short run in the text.

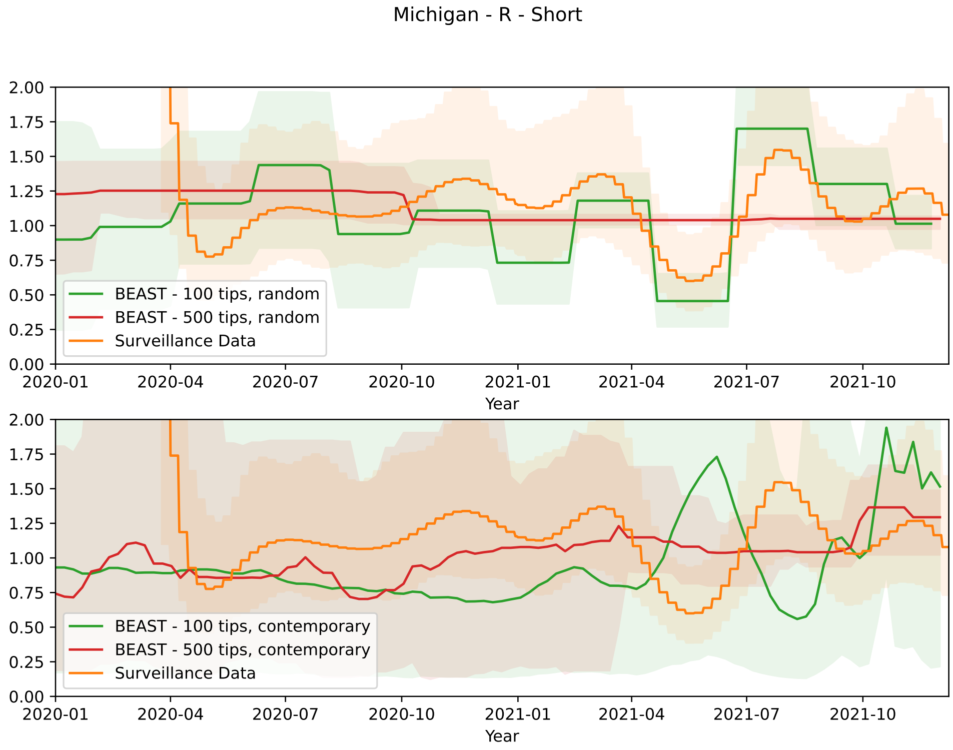

Figure S12: The posterior median and equal-tailed 95% credible interval of R for Michigan given by BEAST. The sampler was allowed to run as long as it VBSKY to analyze the Michigan data. This is referred to as the short run in the text.

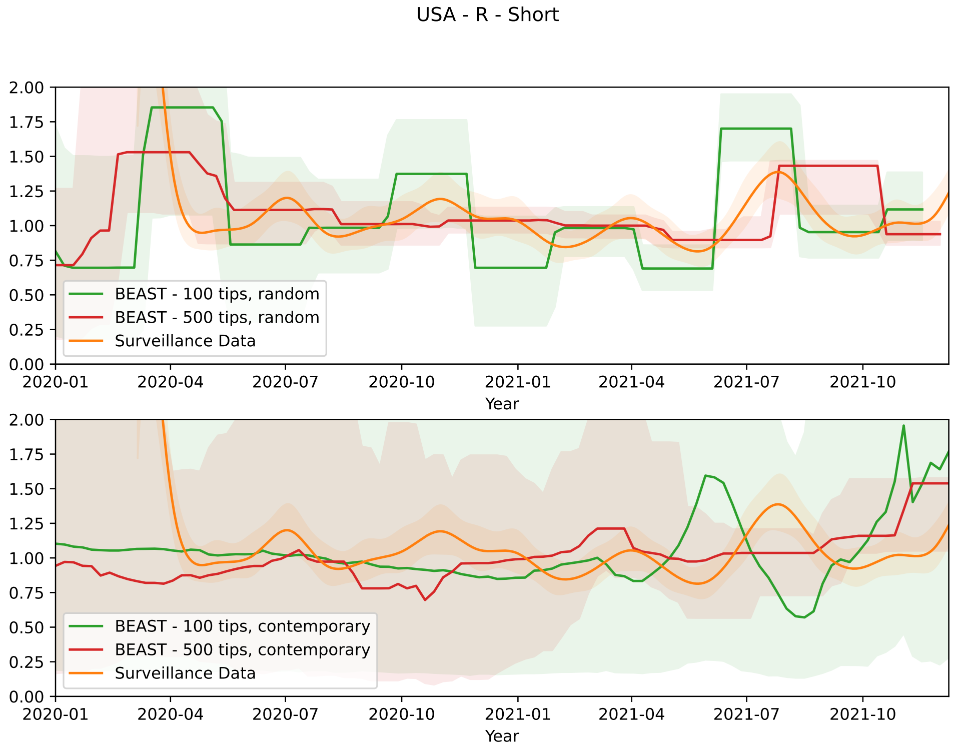

Figure S13: The posterior median and equal-tailed 95% credible interval of R for the USA given by BEAST. The sampler was allowed to run as long as VBSKY to analyze the USA data. This is referred to as the short run in the text.

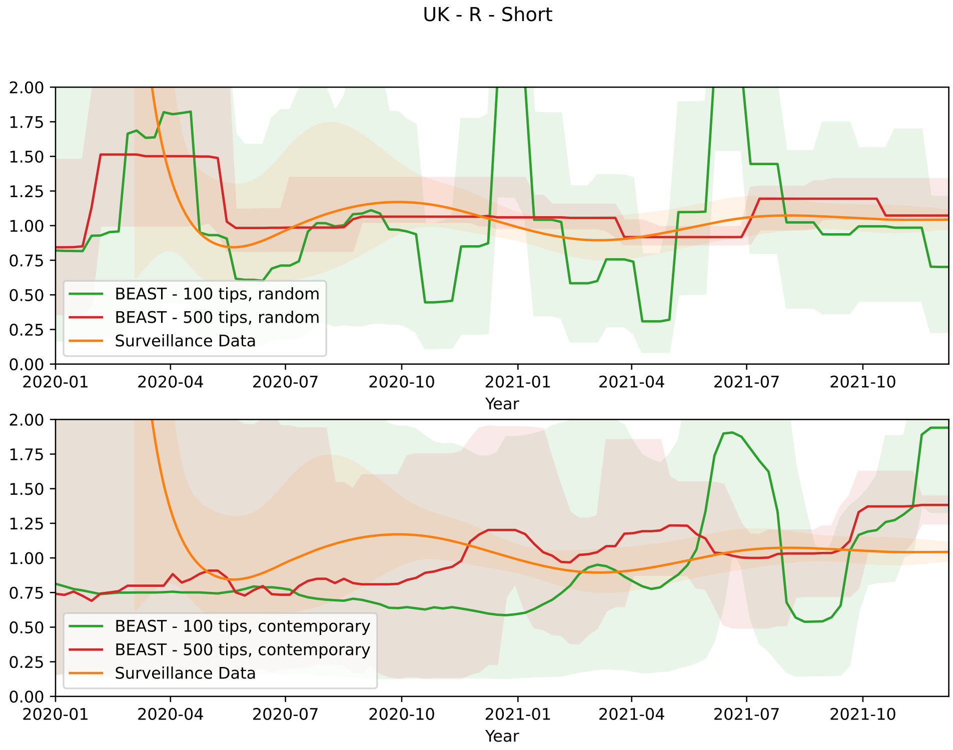

Figure S14: The posterior median and equal-tailed 95% credible interval of R for the UK given by BEAST. The sampler was allowed to run as long as VBSKY to analyze the UK data. This is referred to as the short run in the text.

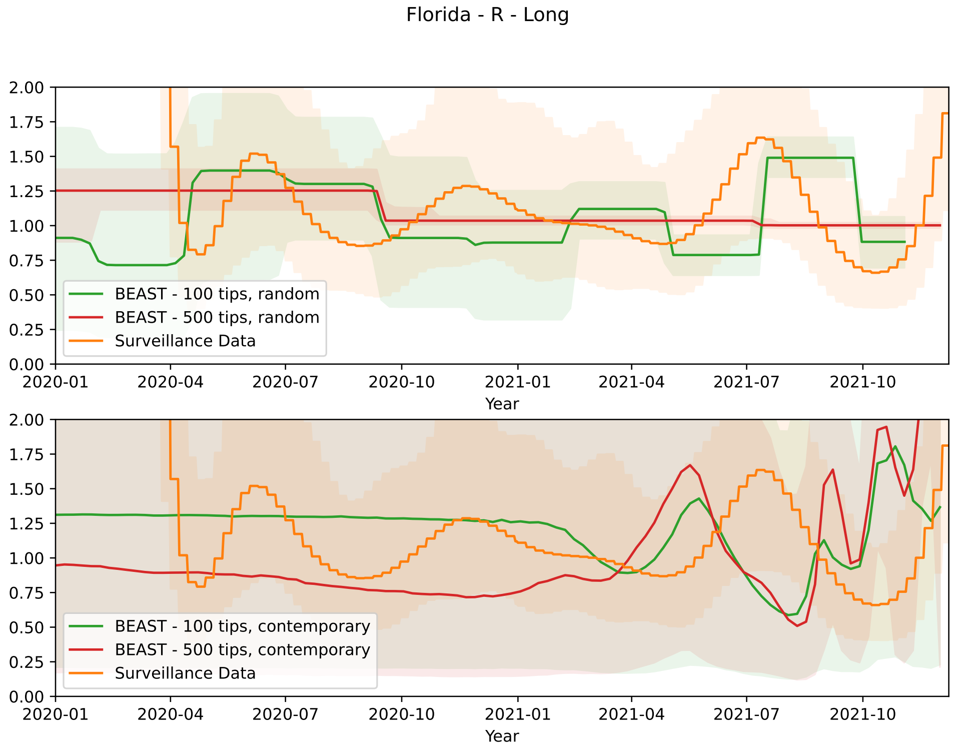

Figure S15: The posterior median and equal-tailed 95% credible interval of R for Florida given by BEAST. The sampler was allowed to run for 100 million steps or 24 hours to analyze the data. This is referred to as the long run in the text.

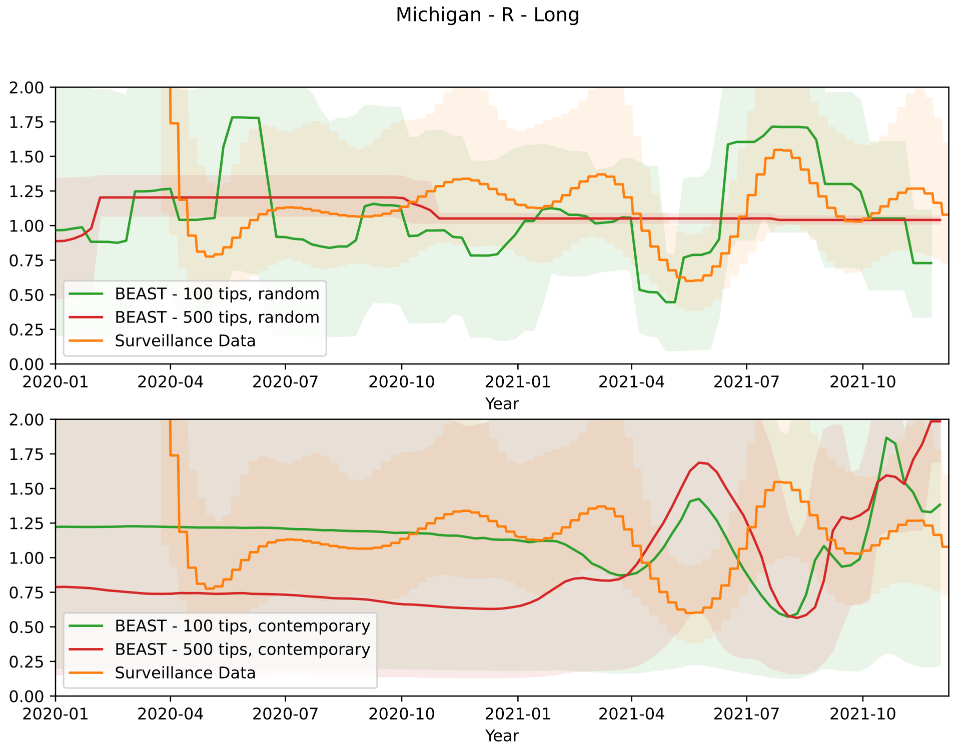

Figure S16: The posterior median and equal-tailed 95% credible interval of R for Michigan given by BEAST. The sampler was allowed to run for 100 million steps or 24 hours. This is referred to as the long run in the text.

USA - R - Long

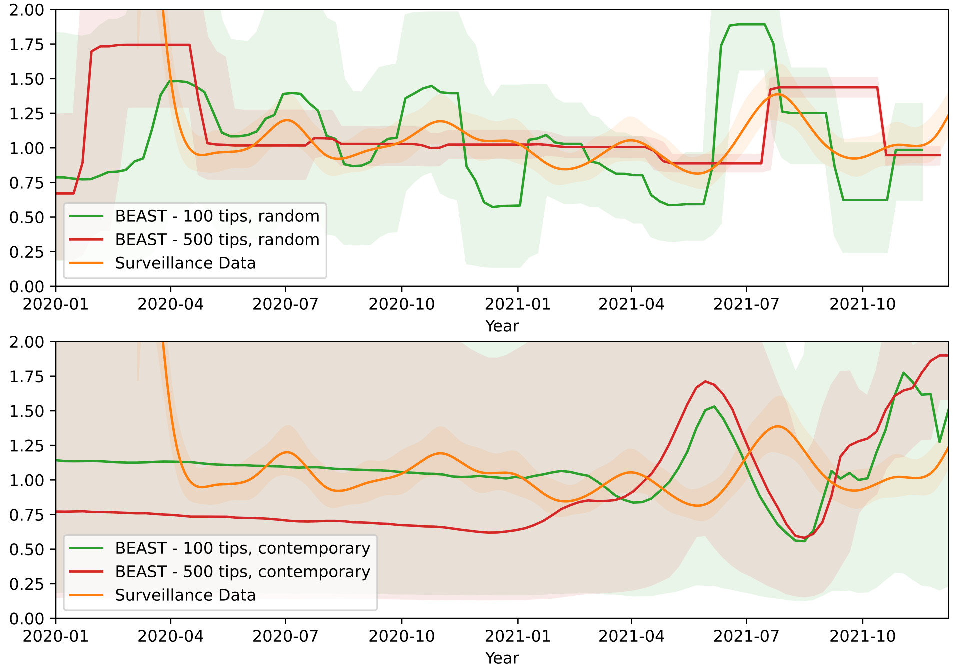

Figure S17: The posterior median and equal-tailed 95% credible interval of R for the U.S. given by BEAST. The sampler was allowed to run for 100 million steps or 24 hours. This is referred to as the long run in the text.

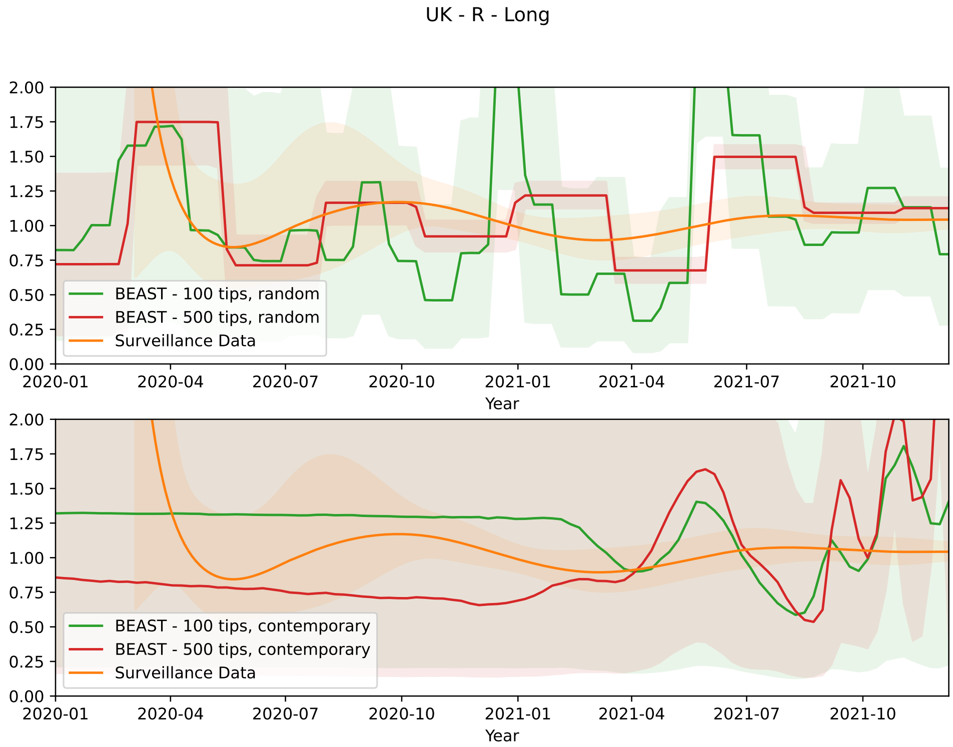

Figure S18: The posterior median and equal-tailed 95% credible interval of R for the UK given by BEAST. The sampler was allowed to run for 100 million steps or 24 hours. This is referred to as the long run in the text.

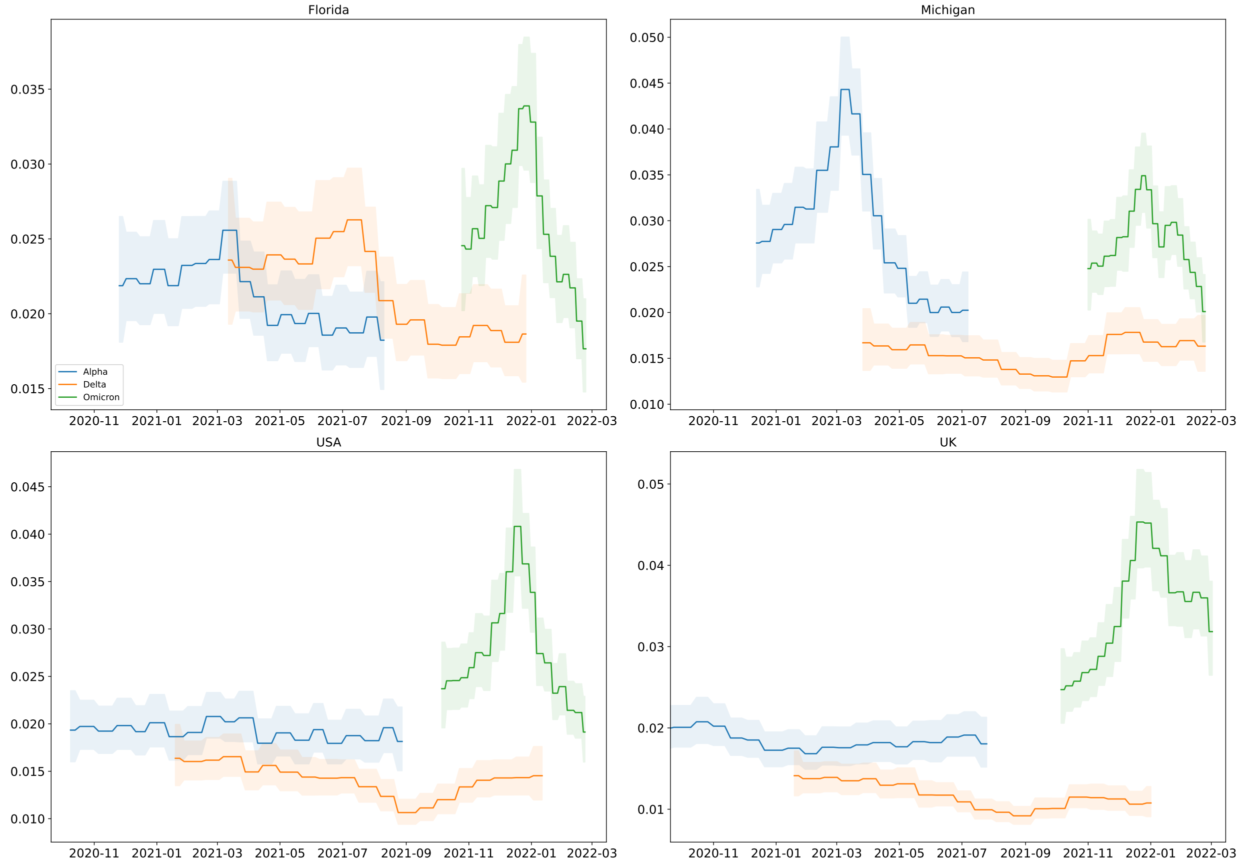

Figure S19: The posterior median and equal-tailed 95% credible interval of s for the Alpha, Delta, and Omicron variants extracting subtrees from the global phylogeny provided by GISAID.

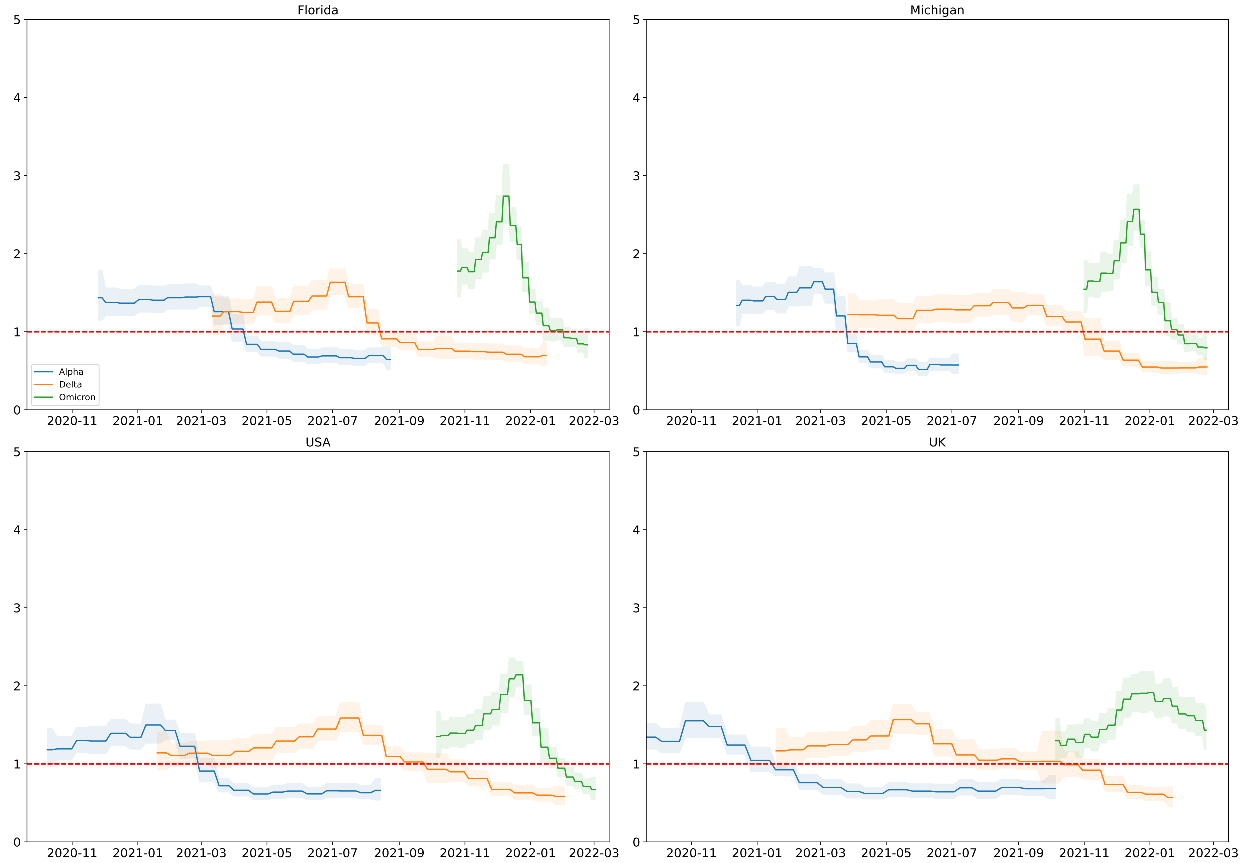

Figure S20: The posterior median and equal-tailed 95% credible interval of R for the Alpha, Delta, and Omicron variants using sUPGMA to estimate tree topologies.

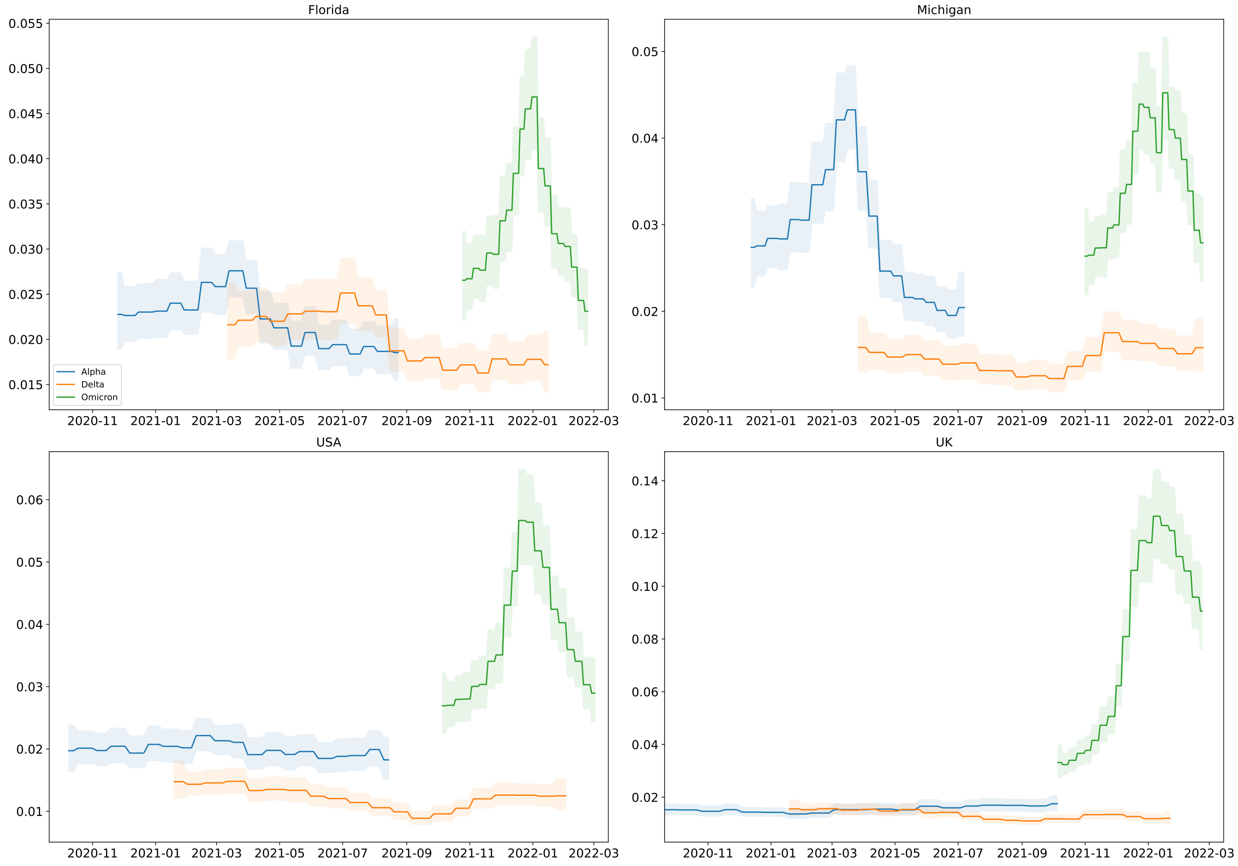

Figure S21: The posterior median and equal-tailed 95% credible interval of s for the Alpha, Delta, and Omicron variants using sUPGMA to estimate tree topologies.

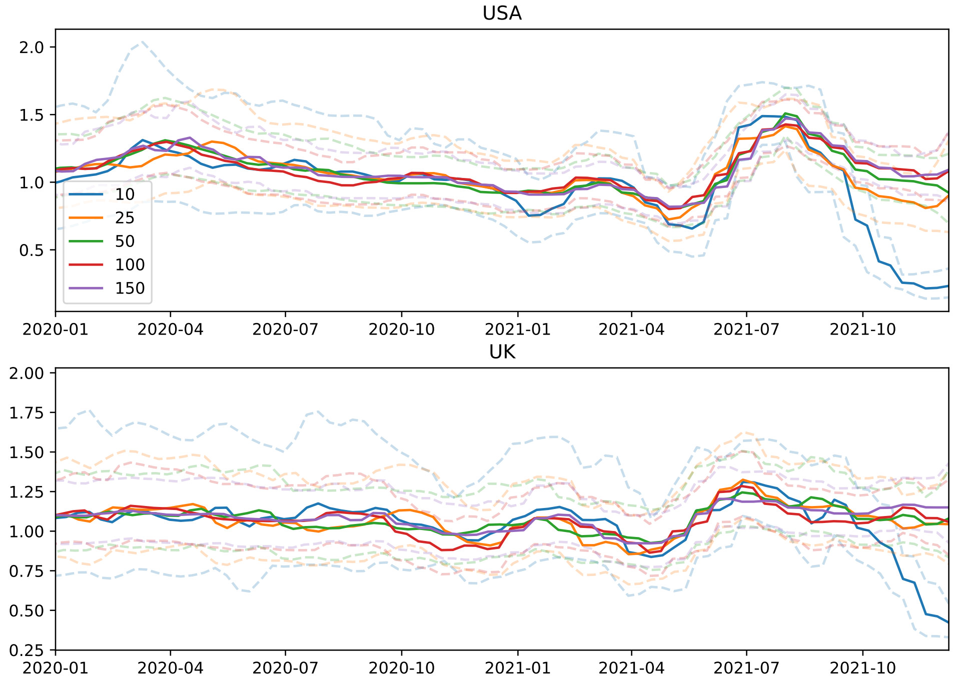

Figure S22: Posterior of $\boldsymbol{R}$ while varying the number of trees. Solid lines represent the median and the dotted lines represent the equal-tailed 95% credible intervals.

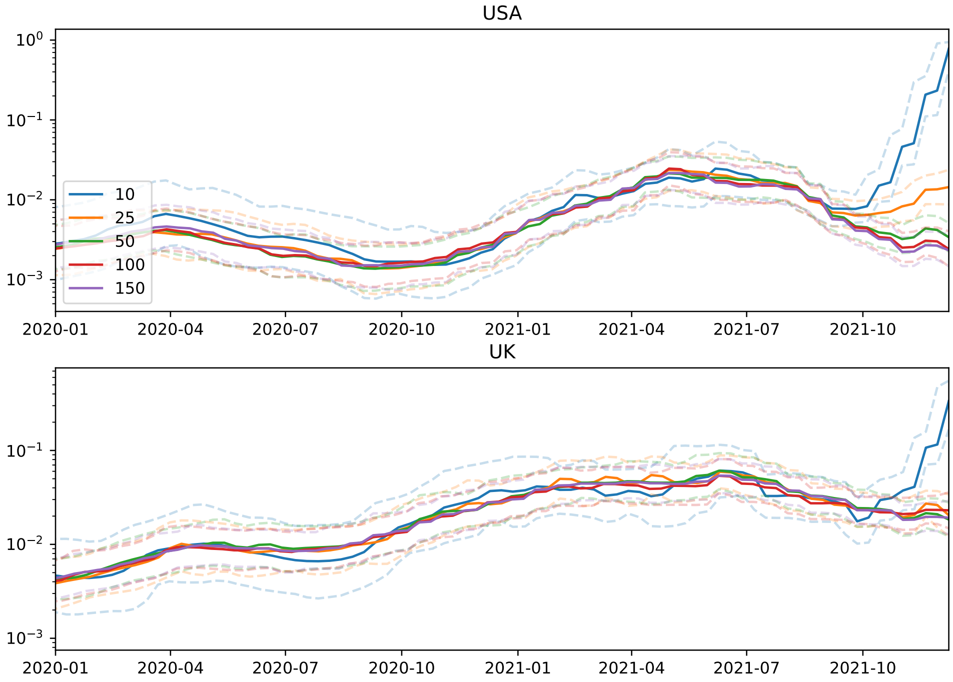

Figure S23: Posterior of $\boldsymbol{s}$ while varying the number of trees. Solid lines represent the median and the dotted lines represent the equal-tailed 95% credible intervals.

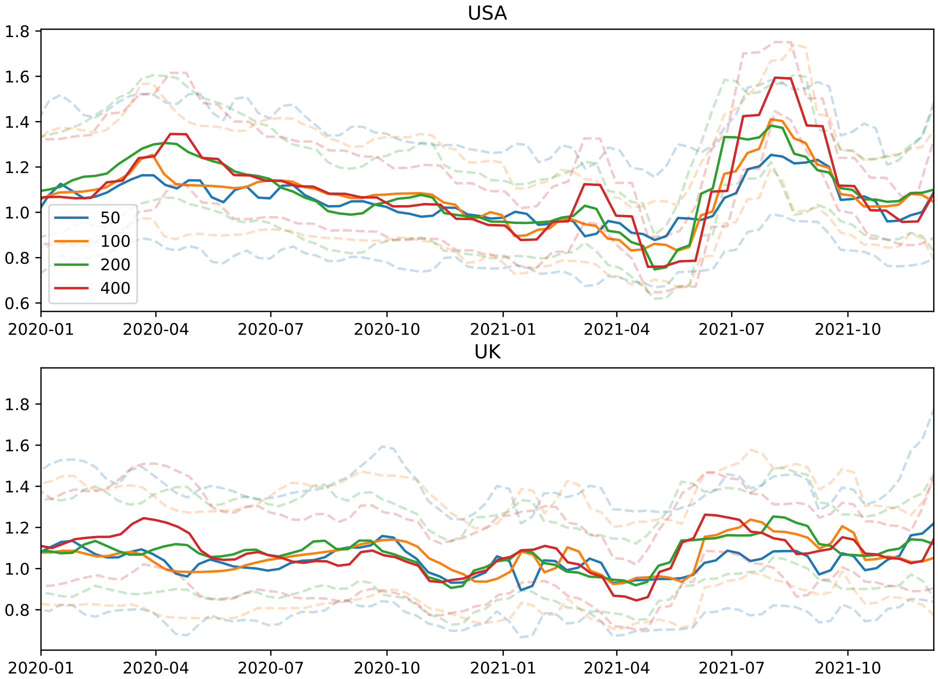

Figure S24: Posterior of $\boldsymbol{R}$ while varying the number of tips. Solid lines represent the median and the dotted lines represent the equal-tailed 95% credible intervals.

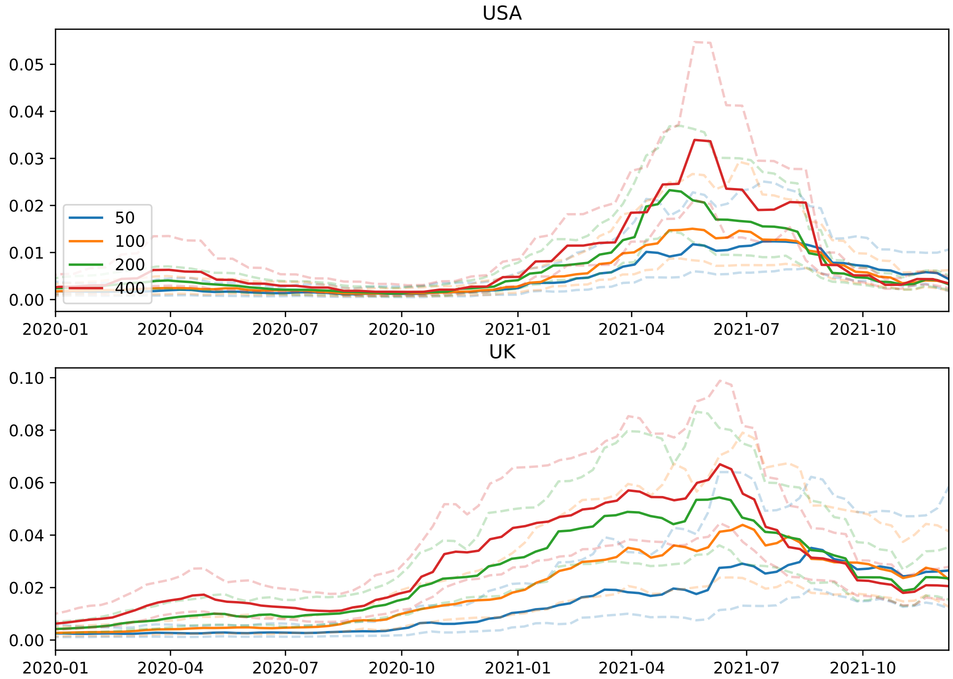

Figure S25: Posterior of $\boldsymbol{s}$ while varying the number of tips. Solid lines represent the median and the dotted lines represent the equal-tailed 95% credible intervals.
